# Preliminary Investigations into the Possibility of Using Hot Torsion Testing to Model Microstructural Evolution During Aluminum Extrusion

**DOI:** 10.3390/ma19194210

**Published:** 2026-10-02

**Authors:** Piotr Korczak, Konrad Błażej Laber, Sonia Boczkal, Dariusz Leśniak, Krzysztof Remsak

**Affiliations:** 1 Łukasiewicz Research Network, Institute of Non-Ferrous Metals, 19 Piłsudskiego Street, 32-050 Skawina, Poland; sonia.boczkal@imn.lukasiewicz.gov.pl (S.B.);; 2 Department of Metallurgy and Metal Technology, Faculty of Production Engineering and Materials Technology, Czestochowa University of Technology, 19 Armii Krajowej Ave., 42-200 Czestochowa, Poland; konrad.laber@pcz.pl; 3 Department of Materials Science and Non-Ferrous Metals Engineering, Faculty of Non-Ferrous Metals, AGH University of Science and Technology, Adama Mickiewicza 30 Ave., 30-059 Krakow, Poland; dlesniak@agh.edu.pl

**Keywords:** numerical modeling, physical modeling, hot torsion test, extrusion, microstructural evolution, EN AW-6060 aluminum alloy

## Abstract

This paper outlines preliminary findings from studies on the possibility of using hot torsion testing for the physical modeling of microstructural evolution during aluminum alloy extrusion processes. Accordingly, two plastic deformation processes were investigated: high-temperature torsion and extrusion. The studies were conducted within the temperature range characteristic of the extrusion of 16 mm diameter round profiles made of EN AW-6060 aluminum alloy, with different cooling rates after deformation. Physical modeling of both processes was preceded by numerical modeling with the finite element method (FEM) and a detailed analysis of the deformation conditions occurring during both processes. Thanks to the careful selection of thermoplastic forming parameters, comparable values of the strain, strain rate, and temperature of profiles suitable for direct microstructural comparison were obtained. The conducted studies made it possible to determine the influence of the applied thermoplastic forming parameters on the microstructural evolution of the tested material. Finite element method simulations revealed distinct strain gradients in both the hot torsion and extrusion processes. Metallographic analysis of the EN AW-6060 alloy showed a strong dependence of grain size and the degree of recrystallization on the thermoplastic processing temperature and the local strain level. The obtained results confirm that hot torsion testing, combined with the selection of representative regions based on FEM analysis, can serve as a supporting method for modeling microstructural evolution in industrial extrusion processes, provided that the thermomechanical conditions correspond to those occurring in the modeled process.

## 1. Introduction

The modern manufacturing industry places increasing emphasis on maximizing the production efficiency of metal alloy semi-finished products in processes such as extrusion or forging. This trend has led to the frequent use of processing parameters close to the material’s limits, including high temperatures, elevated strain rates, and high degrees of deformation [1,2,3]. In recent decades, this approach has become possible to a large extent thanks to the development and widespread use of advanced numerical methods, based primarily on the finite element method (FEM). These tools enable extensive optimization of process parameters while significantly reducing the need for costly and time-consuming physical tests [4,5,6,7,8].

Ongoing trends in process design aim at further improvement of the accuracy of numerical simulations in order to deliberately minimize the safety margins used in industrial production. Such an approach is particularly important in the design and optimization of extrusion and forging processes used to produce components and semi-finished products from aluminum alloys. Reliable numerical simulations require accurate material models that describe the thermomechanical behavior of the alloy under various conditions of thermoplastic deformation [9,10,11].

Currently, the most common experimental methods for developing and calibrating constitutive material models for high-temperature plastic deformation are high-temperature torsion, compression, and tensile tests [12,13,14]. These methods have been described in detail in works such as [15,16,17,18,19]. These experimental techniques allow for the application of large strain values (especially in torsion tests) under well-controlled thermomechanical conditions and enable the determination of yield stress characteristics as a function of applied strain, strain rate, and temperature. According to many authors, the most convenient method for determining high-temperature yield curves is the hot torsion test. It allows the yield stress to be determined indirectly, using the strain-hardening hypothesis. This test is widely used in plastometric studies due to the constant stress state during the test—which closely corresponds to pure shear and the absence of friction—as well as the ability to achieve large strains, significantly exceeding those attainable in tensile or compressive tests [15,16,20]. The hot torsion method is particularly advantageous for evaluating the plasticity of hard-to-deform and brittle materials under hot plastic forming conditions. Another advantage of this method is that, during testing, more reliable conditions can be created to maintain a constant strain rate, and sequential (multi-stage) deformation schemes can be modeled in the best possible way [15,21]. For this reason, data obtained from torsion tests are often considered representative of industrial hot-forming processes for metals and are frequently used as input data for numerical simulations of operations such as extrusion or forging [22,23].

In most practical applications, constitutive models must account for the combined effect of several microstructural mechanisms active during high-temperature deformation. Among these is strain hardening, which leads to an increase in the yield stress as plastic deformation increases, requiring higher forming forces to allow deformation to continue. At the same time, dynamic recovery and dynamic recrystallization processes are activated, which counteract strain hardening, often resulting in a significant reduction in yield stress [24,25,26,27,28]. The relative contribution of these competing mechanisms depends to a large extent on deformation conditions, such as temperature, strain rate, and cumulative (total) strain. These mechanisms have a significant impact on the final structure of the deformed material, influencing grain size and shape as well as crystallographic texture. Furthermore, microstructural evolution does not cease immediately upon completion of the deformation process. During subsequent cooling, the elevated temperature and stored deformation energy allow recovery and recrystallization processes to continue until the temperature drops sufficiently to suppress these phenomena.

It should be emphasized that the torsion test and the extrusion process are characterized by fundamentally different stress and strain states. The torsion test primarily involves shear strain and shear stress with a non-uniform distribution of strain along the radius of the specimen. In contrast, the extrusion process is characterized by a complex triaxial stress state and non-uniform deformation paths. Consequently, even when nominally identical thermomechanical parameters—such as temperature, strain rate, and total strain—are applied, differences in microstructural evolution, including the development of crystallographic texture, can be expected. Although a similar degree of recrystallization can initially be assumed under comparable thermoplastic processing conditions, different strain and stress states can lead to significantly different microstructures.

As mentioned above, the hot torsion test is characterized by an uneven distribution of strain, strain rate, and temperature across the cross-section and along the length of the material being twisted. In the cross-section, all of the aforementioned parameters reach their maximum values at the specimen’s surface, while the lowest values of the strain parameters occur along the material’s axis. The effect of this non-uniformity must be accounted for by calculating the strain parameters for the so-called representative radius, within which the strain parameter values correspond to the average values across the cross-section. Variations in strain and strain-rate values resulting from the analytical relationships used to relate equivalent strain to representative strain may lead to different values of the so-called equivalent radius [16]. Typically, the following values are proposed as equivalent radii, where r denotes the radius of the torsion specimen: 0.6 r, 0.67 r, 0.724 r, and 0.75 r [16,17,29]. Numerical modeling of the torsion test, conducted in accordance with actual experimental test conditions, allows, among other things, for the determination of a representative area for potential metallographic analyses. Based on the obtained results, it is also possible to analyze the state of strain and stress throughout the entire volume of the material. In this study, a radius of 0.6r was adopted as the representative analysis area, corresponding to the average value of true strain across the specimen’s cross-section, which was also confirmed by the results of numerical modeling.

An equally important issue in the analysis of hot torsion tests is determining the influence of the applied deformation parameters on, among other things, the grain size distribution and microhardness of the tested material. According to the literature, both grain size and the nature of its distribution, as well as the distribution of microhardness, depend primarily on the properties of the material itself, as well as on the applied deformation process parameters (set strain value, strain rate, temperature) and the mechanisms (processes) of microstructural transformation [30,31,32,33]. A description of the most common microstructural transformation mechanisms and the behavior of various materials during hot forming can be found, among others, in [34,35,36,37,38,39].

In this study, a detailed comparative analysis was conducted of the crystallographic structures of EN AW-6060 aluminum alloy specimens subjected to multi-variant high-temperature torsion tests and of specimens obtained in an actual extrusion process. A key aspect of the research was conducting physical extrusion tests under conditions strictly corresponding to the selected torsion variants, particularly in terms of the degree/magnitude of deformation, thermoplastic processing temperature, strain rate, and controlled cooling rate after deformation was complete. This approach enables a critical assessment of the suitability of torsion-based material data for the physical modeling of extrusion processes and provides deeper insight into the role of the deformation method in microstructure evolution.

The available technical literature includes studies in which the torsion test was used, among other things, to predict extrusion force [40]. There are also studies in which this test was used for the physical modeling of, among other things, rolling processes (in some cases using variable strain path) [41,42,43]. In the literature analyzed by the authors, no studies were identified concerning the use of hot torsion testing for the physical modeling and assessment of microstructural evolution during extrusion processes, particularly for EN AW-6060 aluminum alloy, combined with verification under semi-industrial conditions. No studies were also identified that analyzed, in this context, the effect of the applied deformation parameters on grain size distribution and microhardness of the investigated alloy. Therefore, the present study extends the existing applications of hot torsion testing to the physical modeling of metal forming processes.

## 2. Materials and Methods

### 2.1. Material Preparation

The study began with the preparation of billets. The EN AW-6060 aluminum alloy was selected as one of the most commonly used alloys in extrusion processes. After preparing the charge in the appropriate proportions, the metal was melted and prepared for casting. After melting the entire charge and reaching a temperature of 735–745 °C, slag was removed from the surface of the metal. Specimens were then taken for chemical analysis, after which the alloy composition was adjusted to meet the requirements of EN AW-6060 [44]. Additionally, grain refiners in the form of TiB_2_ rods were added to achieve a boron content of 30 ppm. After achieving the desired chemical composition (Table 1) and measuring the hydrogen content using an Alscan^®^ device (ABB Measurement & Analytics, Quebec City, QC, Canada), the metal was refined with argon under the following conditions: a refining time of 10 min and gas flow rate of 10 L/min.

**Figure 1:**
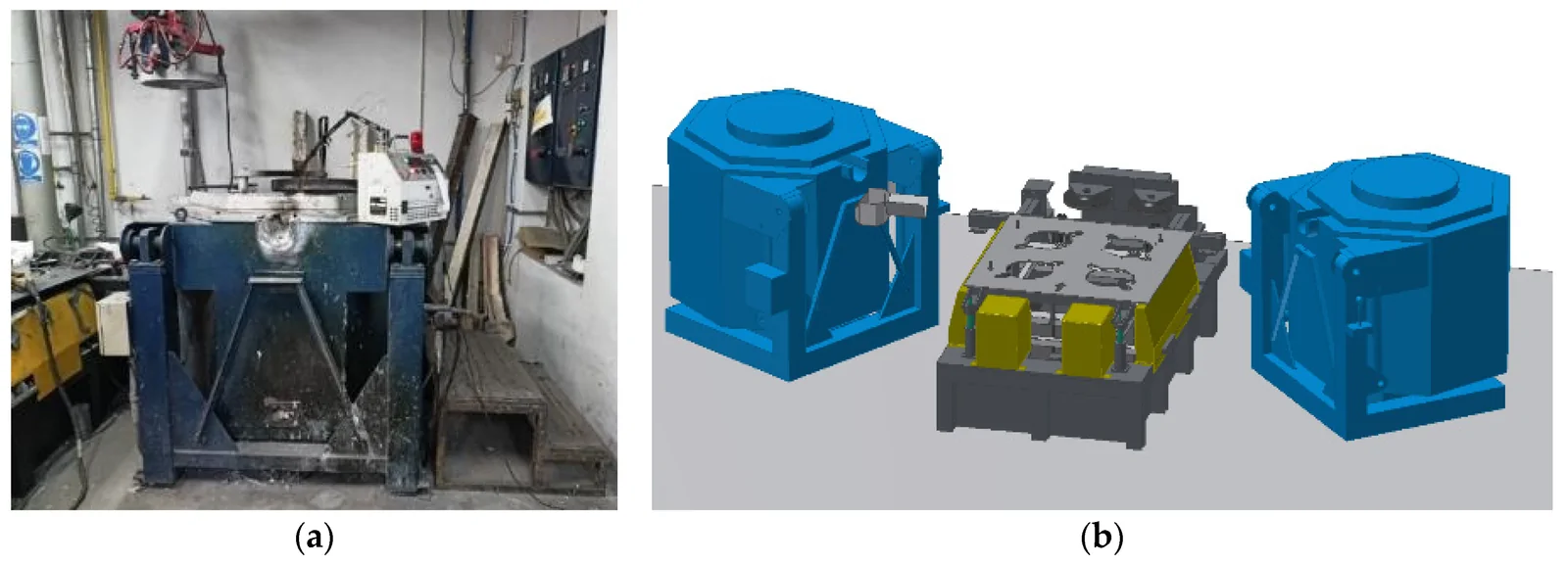
Resistance furnace (Monometer) with a capacity of 300 kg of aluminum (**a**), and experimental line for semi-continuous casting of aluminum billets (**b**).

**Figure 2:**
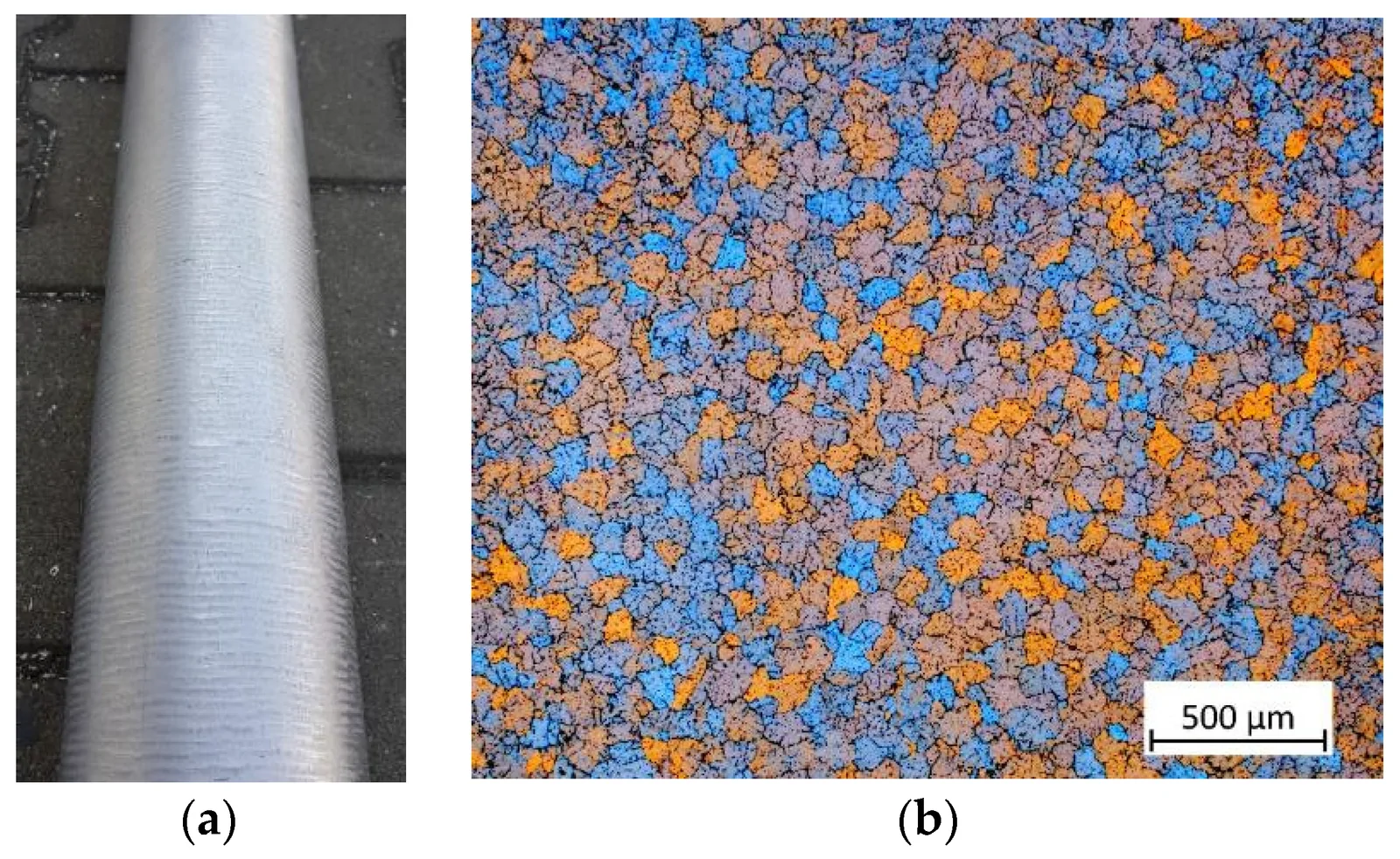
Example of the surface of a cast billet (**a**), and example of microstructure of a cross-section—taken from 2/3 of the billet radius (**b**).

Next, a casting process was carried out to produce billets with a diameter suitable for a semi-industrial extrusion process (102 mm diameter). The casting was performed on a semi-continuous casting line (Figure 1) using two Hot-Top crystallizers with continuous lubrication, each with a diameter of 102 mm. The cast billets, with a diameter of 102 mm, were free of defects such as cracks or tears and had a smooth surface. The billets were subjected to ultrasonic testing. They were then cut into 1.2 m long sections, and 10 mm thick specimens were taken from the center of each billet for structural analysis, and from the beginning and end for evaluation of chemical composition homogeneity. Standard tests, as specified in the foundry’s internal procedures, confirmed the correct structure, composition, and quality of the billets (Figure 2). Homogenization was performed in a LAC^®^ chamber furnace (LAC s.r.o., Židlochovice, Czech Republic) under the following conditions: heating up to 560 °C for 2 h, holding at 560 °C for 5 h, and cooling at a rate of approximately 500 °C/h using a stream of air from an industrial blower. After homogenization, the billets were mechanically machined to a diameter of 96 mm, as required for the 5 MN horizontal press.

### 2.2. Numerical Modeling

Numerical modeling with the finite element method (FEM) for both analyzed processes (high-temperature torsion and extrusion) was performed using QForm Extrusion^®^ software ver. 11 (Micas Simulations Ltd., Oxford, UK). The objective of the numerical modeling was to identify regions characterized by comparable deformation parameters (strain value, strain rate, and temperature). The numerical analysis enabled the selection of representative regions for microstructural studies and a direct comparison of the torsion tests with semi-industrial extrusion conditions performed on a 5 MN press.

The QForm Extrusion^®^ software uses finite element formulas developed specifically for metal forming simulations. In processes involving continuous material flow through the deformation zone, such as extrusion, the Euler model or any Lagrange–Euler (ALE) model is typically used. In contrast, the Lagrange formulation is typically used in volumetric forming processes characterized by local deformation, such as torsion or compression. This approach enables accurate prediction of local distributions of strain, strain rate, and temperature, allowing for reliable identification of regions subjected to comparable thermomechanical conditions.

The friction model used in QForm allows taking into account the adhesion effect between aluminum and steel. In the simulations carried out on the contact surface of the deformed metal and the die, the friction model defined by Levanov [45] was adopted.

(1) $$F_{t}=m\cdot \frac{\sigma_{p}}{\sqrt{3}}\cdot \left(1-e^{\left(-1.25\cdot \frac{\sigma_{n}}{\sigma_{p}}\right)}\right)$$

where: m—friction factor; σ_n_—normal contact pressure; σ_p_—yield stress.

The equation can be considered a combination of the constant friction model and the Coulomb friction model. The second term in parentheses in Equation (1) takes into account the effect of normal pressure on the surface contact. For high pressure values, the expression approximates the conditions specified by the constant friction model. For low pressure values, on the other hand, it defines a linear approximation dependent on the normal stress at the surface contact.

Numerical modeling of high-temperature torsion and extrusion processes was based on material data obtained from previous experimental studies aimed at accurately determining the rheological properties of the tested alloy using the same STD 812 torsion plastometer and the same specimen geometry. After approximating the plastometer test results, the coefficients of the constitutive equation describing the yield stress values of the tested aluminum alloy were determined and implemented in the numerical model to describe changes in yield stress as a function of strain, strain rate, and temperature.

To describe the changes in yield stress during the approximation of the experimental results, the Hensel–Spittel function was adopted in the form implemented in the QForm Extrusion^®^ software (2) [46]:

(2) $$\sigma_{p}=A\cdot e^{m_{1}\cdot T}\cdot T^{m_{9}}\cdot \varepsilon^{m_{2}}\cdot e^{\frac{m_{4}}{\varepsilon }}\cdot {\left(1+\varepsilon \right)}^{m_{5}\cdot T}\cdot e^{m_{7}\cdot \varepsilon }\cdot {\dot{\varepsilon }}^{m_{3}}\cdot {\dot{\varepsilon }}^{m_{8}\cdot T}$$

where: $\sigma_{p}$—yield stress; T—temperature; ε—true strain; $\dot{\varepsilon }$—strain rate; A;material cinstant m_1_ ÷ m_9_—function coefficients determined experimentally.

The material constants (Table 2) were identified based on data from plastometric tests using the built-in solver of the QForm Extrusion^®^ software.

**Figure 3:**
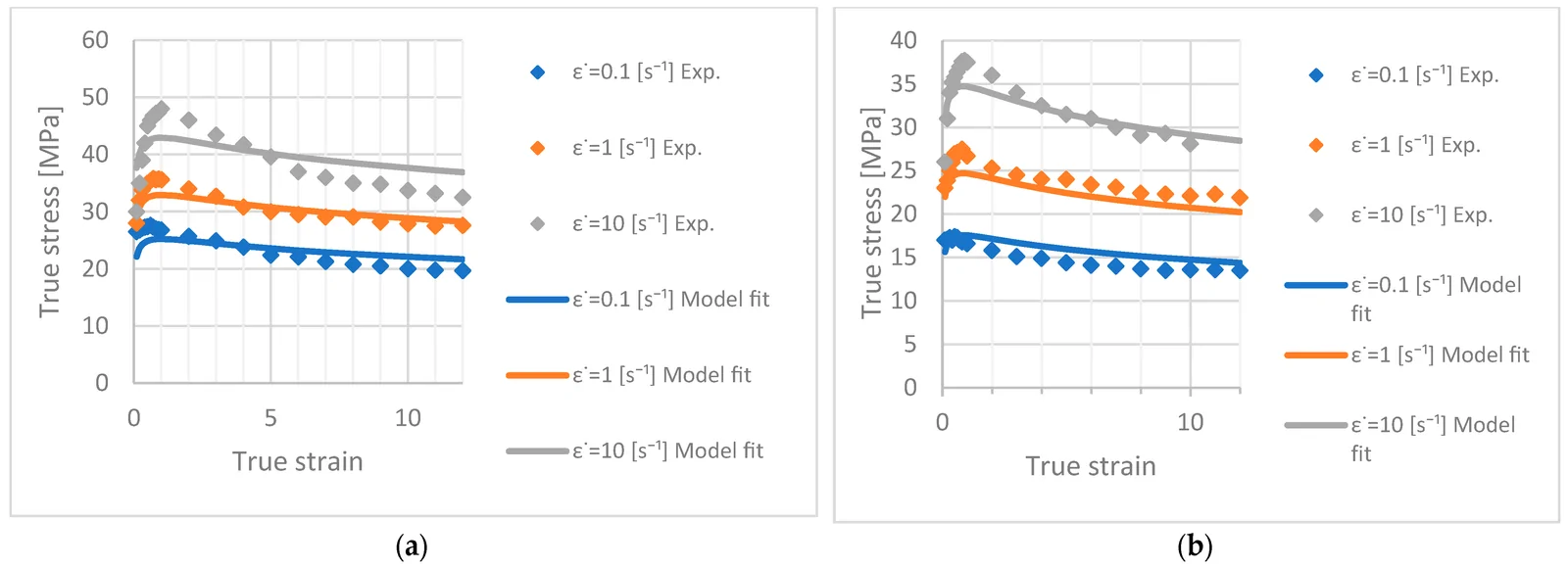
Example yield curves for the EN AW-6060 alloy obtained from torsion tests at 450 °C (**a**) and 500 °C (**b**), along with approximate values calculated using the Hensel–Spittel equation.

Exemplary plastic flow curves for the EN AW-6060 alloy obtained from torsion tests, along with approximate values calculated using the Hensel–Spittel equation and the coefficients listed in Table 2, are shown in Figure 3.

Based on an analysis of the data presented in Figure 3, it was found that, within the examined range of strain parameters, the yield stress values of the tested aluminum alloy obtained by approximation using Equation (2) and the coefficients listed in Table 2 are close to the actual values obtained during hot torsion tests. The accuracy of the model fit was assessed based on error metrics provided by the QForm Extrusion equation-solving software during the parameter identification procedure. The average error between the experimental and approximated values was approximately 6%, and the maximum error did not exceed 27%. The achieved level of agreement confirms that the identified parameters of the Hensel–Spittel equation provide a satisfactory representation of the material’s behavior and can be used in further numerical simulations of torsion and extrusion processes.

The studies presented in this paper (numerical modeling, physical modeling, and verification under semi-industrial conditions) were conducted within the temperature range characteristic of the extrusion process of 16 mm diameter round rods made of EN AW-6060 aluminum alloy, with various cooling rates following deformation (Table 3). These parameters are representative of the thermomechanical conditions during the semi-industrial extrusion process analyzed in this study. Additionally, the capabilities of the STD 812 torsional plastometer were taken into account with regard to its ability to obtain the required values of deformation parameters and cooling rates after the deformation process is complete. Numerical simulations were performed to determine the distribution and gradient of deformation parameters in the cross-sections of the specimens for both the extrusion and torsion processes. The FEM analysis of the extrusion process showed that the local true strain within the extruded profile ranged approximately from 3 to 6. Therefore, a target true strain of 5 was selected for the torsion tests as a representative deformation level for the comparative analysis. The simulation parameters were selected to achieve the previously defined conditions representative of the actual extrusion process.

#### 2.2.1. Numerical Modeling of the Torsion Test

**Figure 4:**
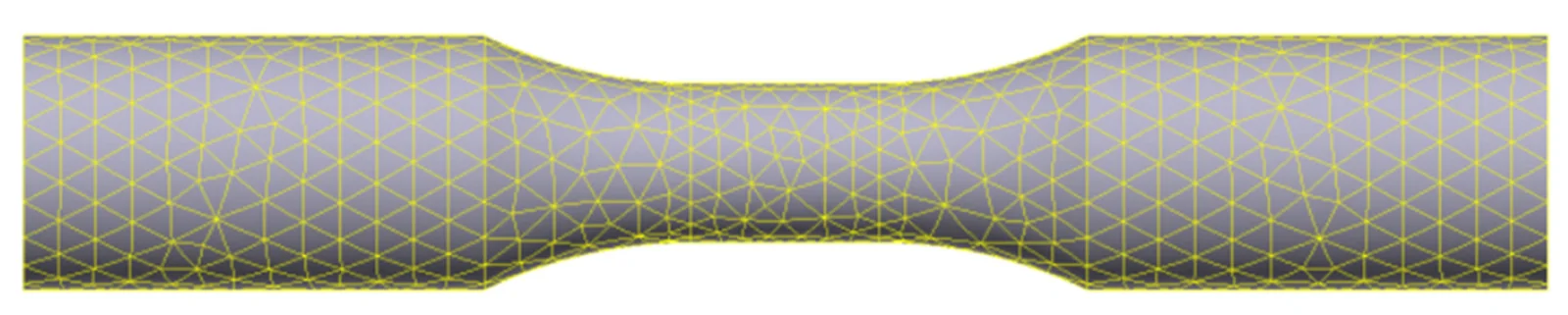
An example of a specimen geometry with a finite element mesh applied for the numerical modeling of a constrained torsion test.

Numerical modeling of the constrained torsion process was performed using a three-dimensional adaptive finite element mesh. The initial mesh consisted of 2730 elements, while after deformation, the number of elements increased to 189,239 as a result of mesh adaptation. A friction coefficient of zero was assumed for the contact between the specimen and the plastometer grips. The initial temperature of both the specimen and the dies was set in accordance with the current test parameters (Table 3). To maintain a constant temperature during numerical simulation, the specimen temperature was taken as the ambient temperature, since temperature measurements taken during physical torsion tests using thermocouples did not show significant temperature changes during the deformation process. Adaptive mesh refinement allowed for the accurate representation of large plastic deformations as well as local strain and temperature gradients. An example of the specimen geometry with a finite element mesh, implemented in the Qform Extrusion^®^ software, is shown in Figure 4.

#### 2.2.2. Numerical Modeling of the Extrusion Process

**Figure 5:**
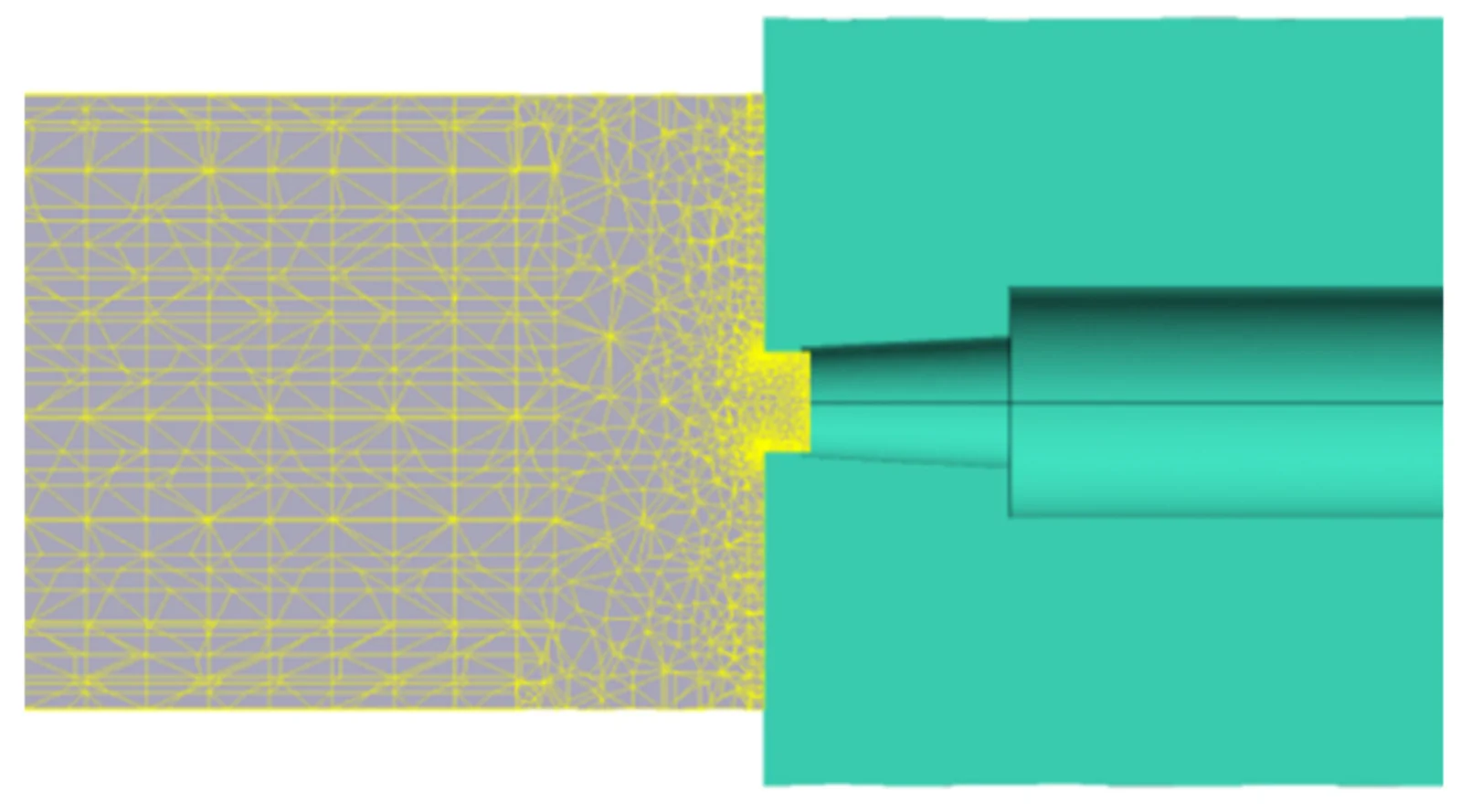
Example geometry of an extruded billet with a finite element mesh applied for numerical modeling of the extrusion process.

The extrusion process was modeled by assuming the same initial temperature for both the billets and the dies (depending on the variant under study—Table 3). Friction at the interface between the billet and the die was described using the Levanov friction model (1), with a friction coefficient of 1 and a Levanov coefficient of 1.25. The initial diameter of the billet was 96 mm, and its length was 300 mm. A three-dimensional adaptive finite element mesh was used, consisting of 161,015 elements at the start of the simulation and increasing to 400,385 elements in the final stage of the process. An example of the billet geometry with the applied finite element mesh, implemented in the Qform Extrusion^®^ software, is shown in Figure 5.

#### 2.2.3. Physical Modeling—Restrained Torsion Test—STD 812 Torsion Plastometer

**Figure 6:**
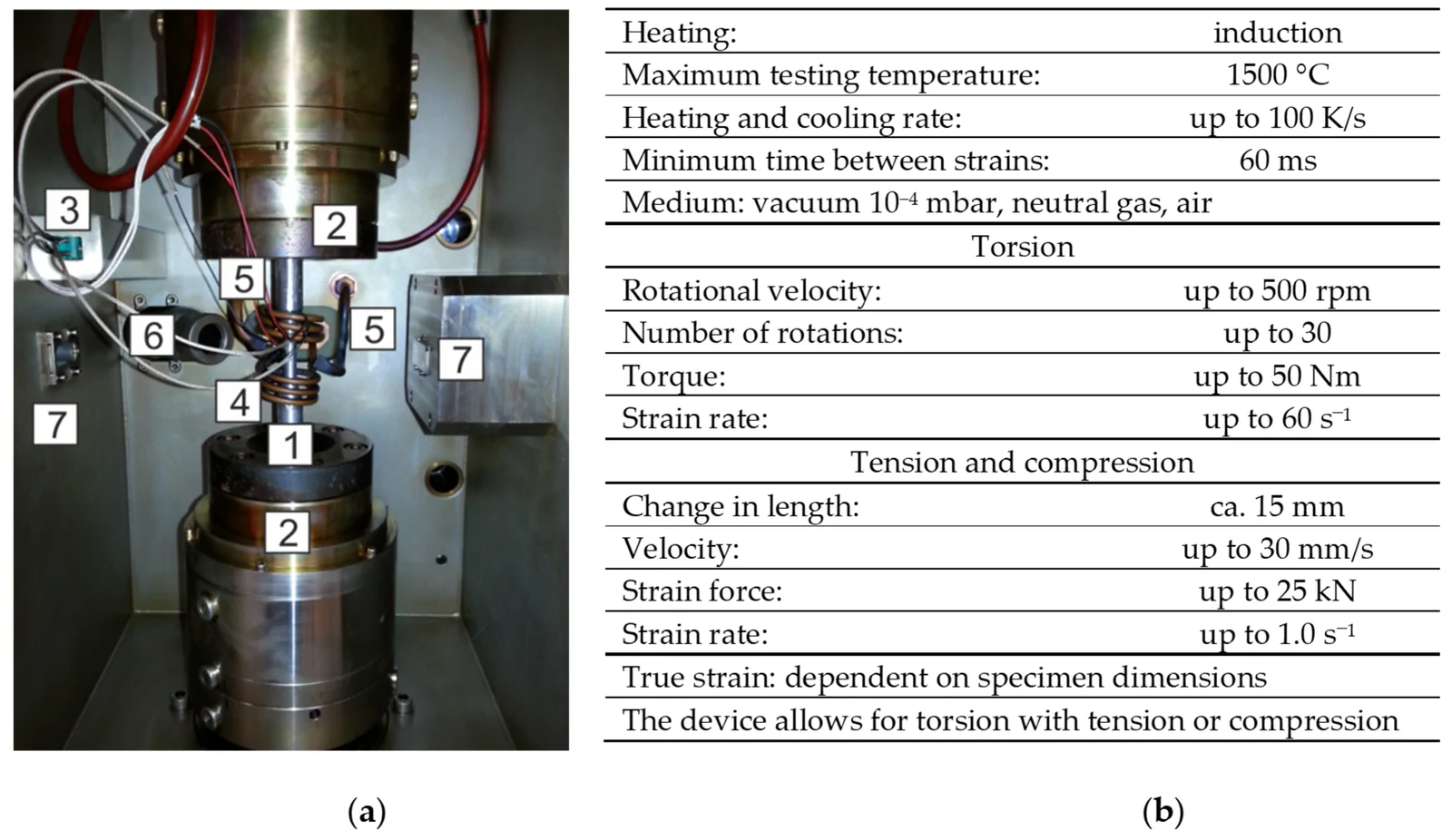
STD 812 torsion plastometer [48]: (**a**) device chamber: 1—specimen; 2—holders; 3—thermocouples type S; 4—induction solenoid; 5—cooling system jets; 6—pyrometer; 7—sensors for laser measurement of specimen diameter; (**b**) basic specification.

Physical modeling of the extrusion process for 16 mm diameter round profiles made of EN AW-6060 alloy was conducted in constrained torsion tests using the STD 812 torsion plastometer [47]. A general view of the test chamber and the main parameters of the device are shown in Figure 6.

**Figure 7:**
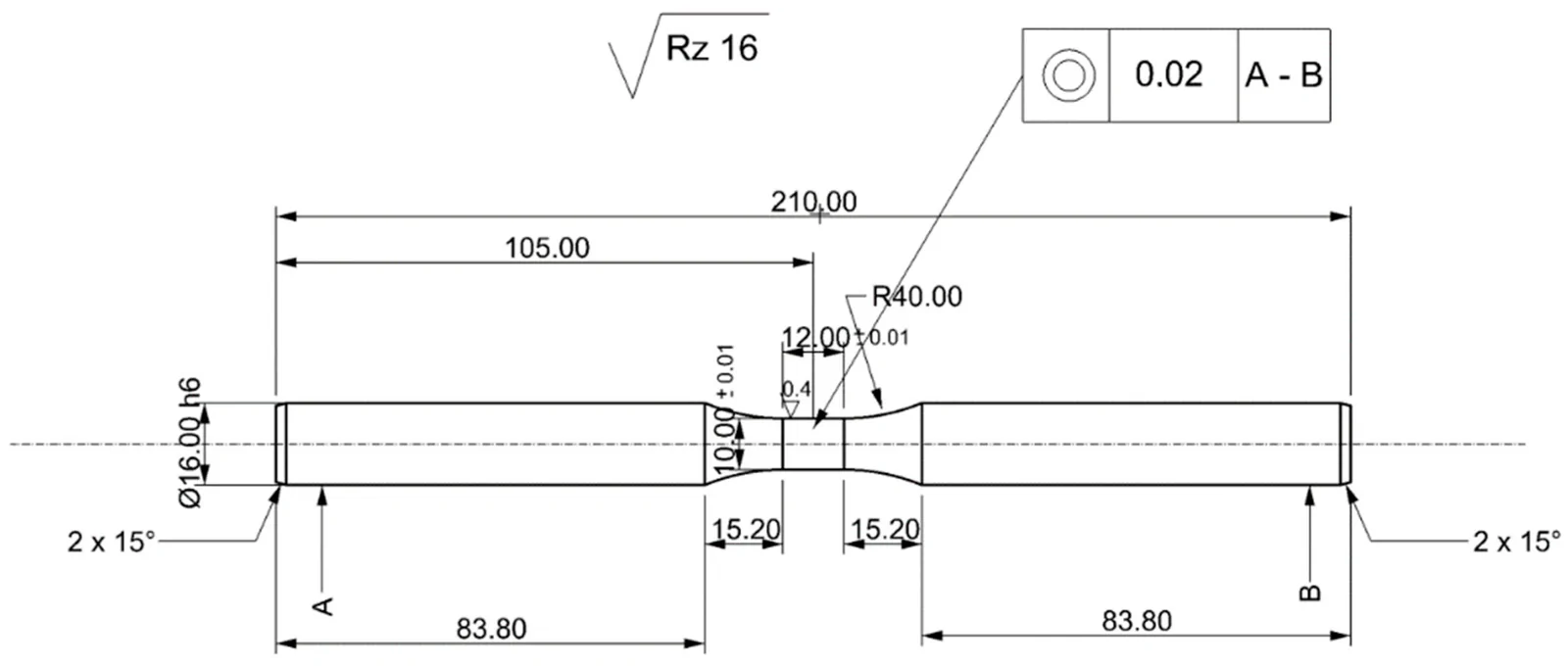
Technical drawing of the specimen for physical modeling of the extrusion process of the EN AW-6060 alloy in constrained torsion tests.

The initial data for the physical modeling of the extrusion process were determined based on numerical modeling (main test parameters—Table 3). The physical modeling was conducted for a true strain value of 5, at a constant temperature of the deformed specimen, and at a constant strain rate of 5 ^s−1^. Three independent specimens were tested for each investigated hot torsion condition. Circular specimens with a diameter of d = 10 mm and a measurement base length of l = 12 mm were used for the tests (Figure 7).

A type K (NiCr-Ni) thermocouple welded to the side surface of the specimen was used to record and monitor temperature changes. The test material was heated at a rate of 5 °C/s to a set temperature (depending on the tested variant—Table 3), held for 300 s, and then deformed and cooled at a rate of 1.7 or 15 °C/s, depending on the variant.

Equation (3) was used to determine the true strain; the true strain rate was determined based on Equation (4); and the yield stress was calculated according to Equation (5) [2,22]:

(3) $$\varepsilon =\frac{2\cdot \pi \cdot r\cdot N}{\sqrt{3}\cdot L}$$

(4) $$\overset{\cdot }{\varepsilon }=\frac{2\cdot \pi \cdot r\cdot \overset{\cdot }{N}}{\sqrt{3}\cdot 60\cdot L}$$

(5) $$\sigma_{p}=\frac{\sqrt{3}\cdot 3M}{2\pi r^{3}}$$

where: r—specimen radius; L—specimen length; N—number of torsions (rotations) of the specimen; $\dot{N}$—torsion rate (rotational velocity); M—torque.

**Figure 8:**
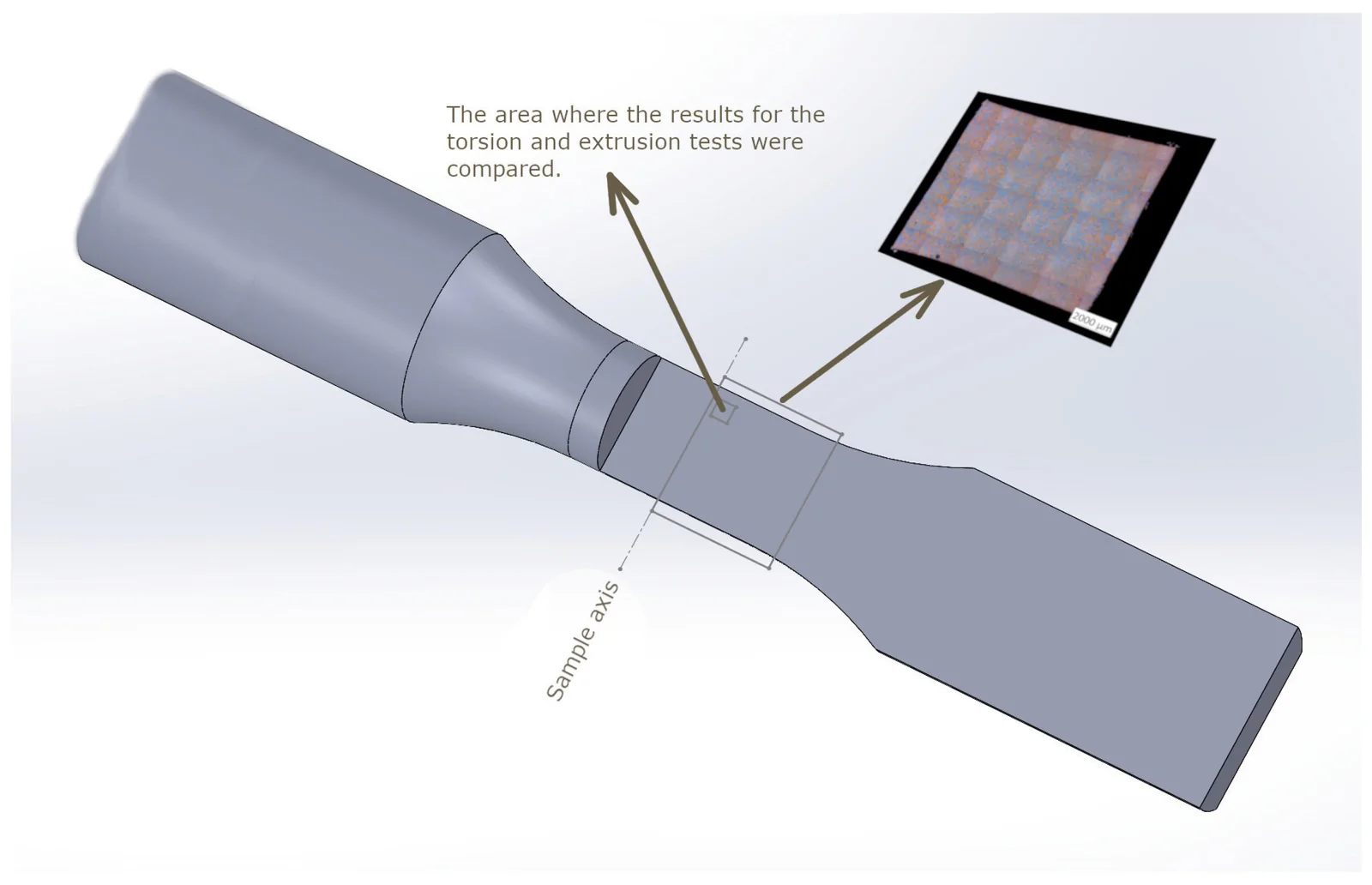
Schematic of specimen extraction for metallographic testing following physical modeling.

After physical modeling, the specimen was cut according to the scheme shown in Figure 8, and specimens were extracted for structural analysis. For each investigated condition, one longitudinal and one transverse section were prepared for metallographic examination. The selection of test areas was based on finite element method (FEM) analyses and the authors’ experimental experience to ensure a microstructural comparison of areas characterized by similar levels of deformation parameters and comparable thermomechanical conditions.

The main advantage of the torsion test is the ability to achieve very high values of true strain (which is difficult or impossible to achieve in tensile or compressive tests) without changing the specimen’s external dimensions. It should be emphasized, however, that this test is characterized by a non-uniform distribution of strain and stress parameters, which directly affects the evolution of the material’s microstructure across the specimen’s cross-section. Consequently, the microstructure after deformation exhibits a distinct radial gradient, which necessitates careful selection of observation areas and a thoughtful approach to the interpretation of microstructural data. Taking these factors into account, this study places particular emphasis on analyzing microstructural changes as a function of radial position in the specimens, enabling a direct correlation between local deformation conditions and the dominant mechanisms of plastic deformation and microstructure evolution.

#### 2.2.4. Extrusion Process Under Semi-Industrial Conditions—5 MN Horizontal Press

**Figure 9:**
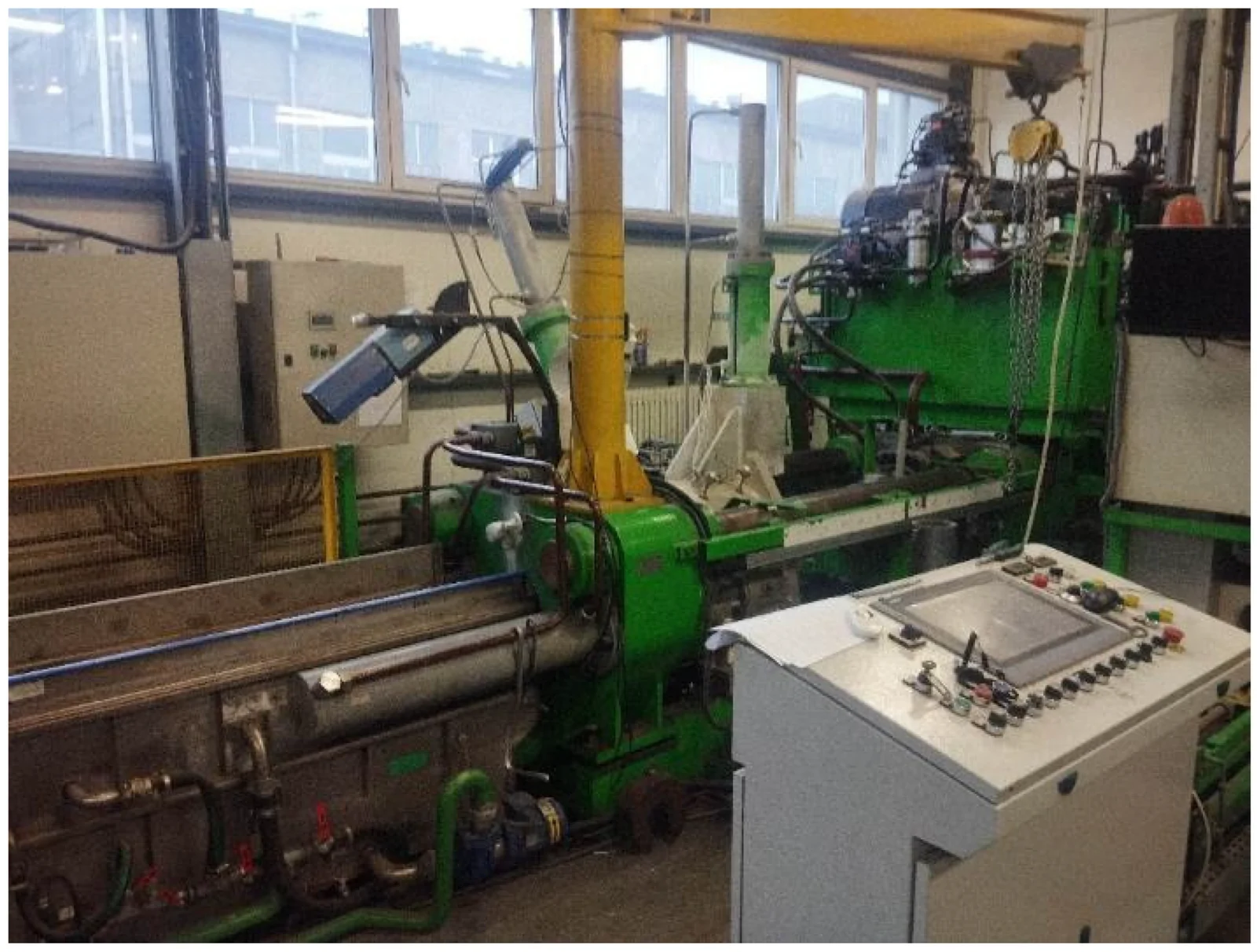
General view of the 5 MN semi-industrial horizontal press manufactured by ZAMET.

**Figure 10:**
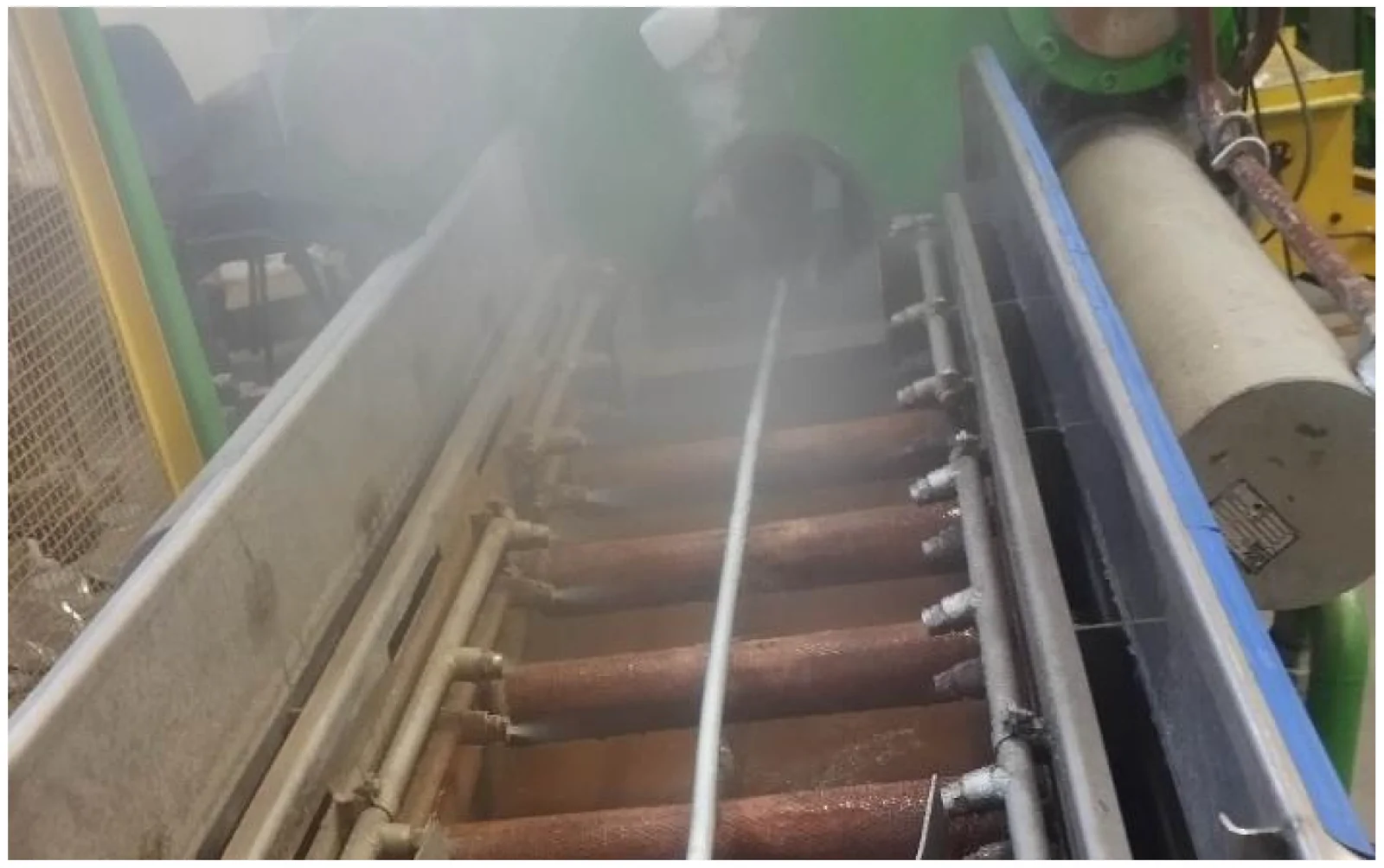
General view of the water mist cooling zone of the 5 MN horizontal press manufactured by ZAMET.

The extrusion experiments were conducted using a semi-industrial horizontal press with a maximum pressing force of 5 MN, manufactured by ZAMET. The press was equipped with an electrically heated container with an inner diameter of 100 mm. The extrusion tests were conducted using a flat single-hole die. Both the billet and the tooling (die and housing) were heated in a resistance furnace. To achieve cooling conditions comparable to those prevailing immediately after plastic deformation during torsion tests, two cooling methods were used: forced air circulation cooling and water mist cooling. The intensity of both cooling systems was adjusted to best reflect the cooling conditions used in the torsion experiments. In the case of forced-air cooling, a cooling rate in the range of 1.5–2 °C/s was achieved, while the use of water mist allowed for a cooling rate of 17 °C/s. Although the higher cooling rate slightly exceeded that achieved in the torsion tests, it represents the lowest cooling intensity available in the press’s cooling system. Representative images from the analyzed extrusion process are shown in Figure 9 and Figure 10.

The extrusion process was conducted at a ram speed of 3 mm/s, selected to provide deformation conditions comparable to those corresponding to a strain rate of 5 s^−1^ in the torsion test. The experiments were conducted at four different temperatures (for both the billet and the dies): 400, 450, 500, and 550 °C (Table 3). During the extrusion process, the temperature of the extruded rod was continuously monitored using an A300 (3T) pyrometer| Accurate Sensors Technologies Ltd. Misgav Industrial Park, Misgav 20174, Israel| designed for aluminum alloys. Specimens were taken from a section of the extruded rod for which temperature measurements confirmed that the temperature had stabilized at the set level. For each investigated extrusion condition, the analyses were performed using material obtained from one semi-industrial extrusion run. The specimens used for metallographic, EBSD, microhardness, and mechanical property analyses were taken from the same stabilized section of the extruded profile and therefore were not treated as independent process replicates. Three tensile specimens were tested for each investigated condition for both the hot torsion and semi-industrial extrusion processes.

## 3. Results and Discussion

### 3.1. Results and Analysis of Numerical Modeling

Numerical modeling with the finite element method was employed to define so-called representative regions and to analyze the distributions of true strain, strain rate, equivalent stress, and temperature during the torsion and extrusion processes. The results obtained served as the basis for correlating local thermomechanical conditions with the observed microstructure and microhardness of the tested material.

Based on the numerical analysis, it was determined that in order to correctly model the physical process of extruding 16 mm diameter round rods and ensure a comparable level of strain parameters, the torsion test should be conducted up to an average true strain value of 5 with an average strain rate of 5 ^s−1^.

**Figure 11:**
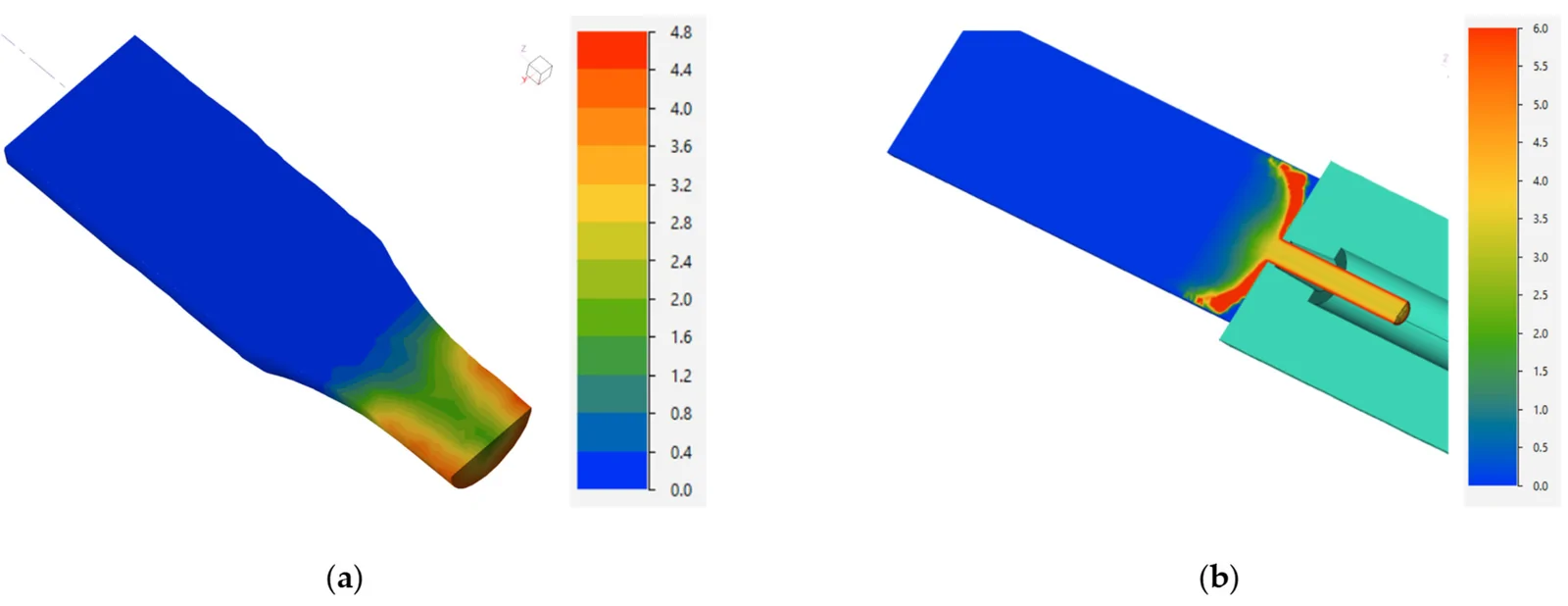
Examples of strain distributions obtained from numerical simulations: (**a**) torsion test, (**b**) actual extrusion process.

Examples of strain distributions in both the extrusion and torsion processes are shown in Figure 11.

Furthermore, analysis of the numerical simulation results revealed a distinct gradient in true strain in both processes (Figure 4 and Figure 5). In the case of the torsion test, the strain gradient was particularly pronounced. The highest values of true strain occurred on the specimen’s surface, while the specimen’s axis remained practically undeformed. In the extrusion process, the true strain gradient in the cross-section was lower and ranged from approximately 3 to 6 within the extruded profile. In this case, higher strain values were observed in the near-surface region, resulting from uneven material flow and the presence of dead zones.

Therefore, an additional analysis of the FEM results was performed for the region located at approximately 0.6r in the torsion specimen and the corresponding region in the extrusion process. The obtained ranges of local temperature, true strain, and strain rate are summarized in Table 4. It is worth noting that Table 3 summarizes the target thermoplastic forming parameters used in the experiments, whereas Table 4 presents the local thermomechanical parameters obtained from FEM for the selected representative regions.

The comparison shows that the true strain and strain rate ranges are closely comparable in the selected regions. The temperature range in the extrusion process is somewhat higher and reflects the temperature increase observed in the corresponding region.

**Figure 12:**
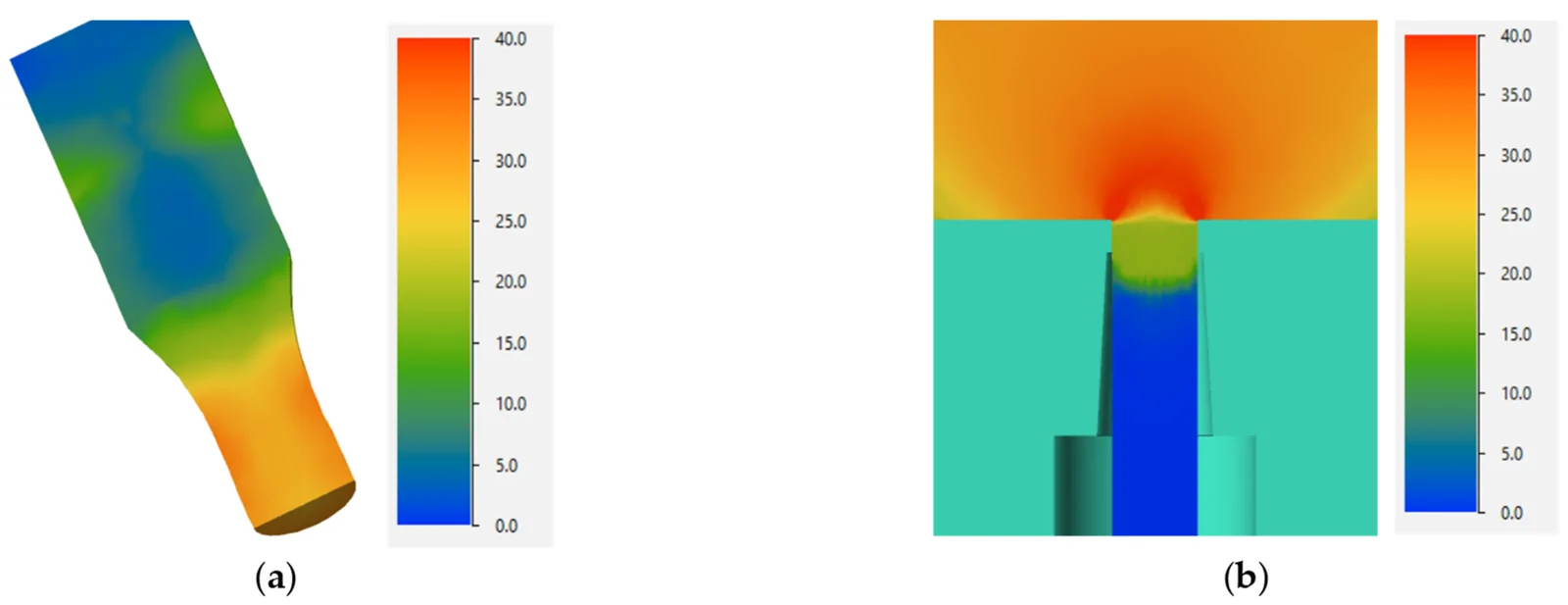
Example stress distributions (in MPa) obtained from numerical modeling—deformation temperature 500 °C: (**a**) torsion test, (**b**) actual extrusion process.

An analysis of the numerical simulation results for deformation processes at 500 °C revealed that in the torsion test, within a representative region located at approximately 0.6 of the specimen’s radius, the equivalent stress was approximately 30–40 MPa. A comparable stress level was observed in the extruded material within the die bearing zone which represents the zone of most intense plastic deformation and the final shaping of the finished product’s profile. While local variations in stress occurred in the feed area, the stress level in the die’s calibration strip area remained similar to that observed in the torsion test (Figure 12).

**Figure 13:**
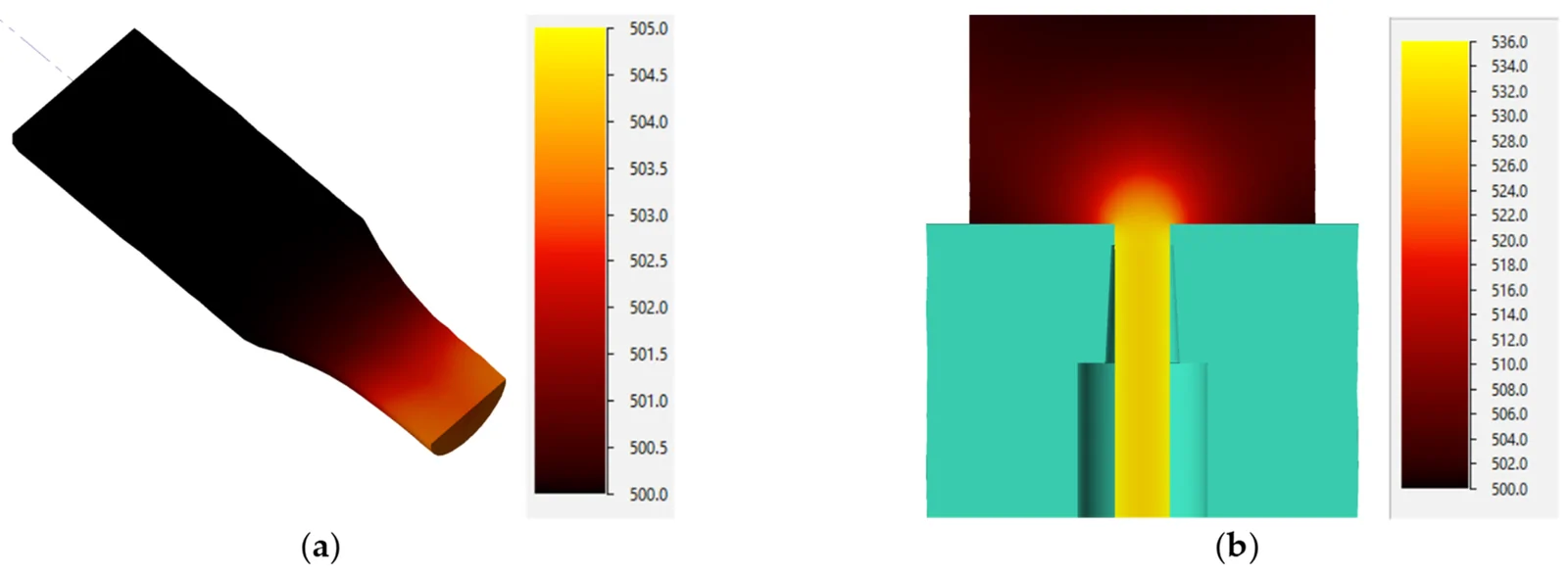
Example temperature distributions obtained from numerical simulations—target deformation temperature of 500 °C: (**a**) torsion tests, (**b**) actual extrusion process.

Analysis of the numerical simulation results shown in Figure 13 revealed that during the torsion test conducted at 500 °C, the temperature field was practically uniform. In a representative region of the specimen, the temperature ranged from approximately 499 to 503 °C. In contrast, a greater temperature gradient was observed during the extrusion process, reaching approximately 30 °C across the cross-section of the extruded profile. Despite this, the temperature in the area of the die’s bearing zones remained close to the nominal process temperature and corresponded to the conditions obtained during the torsion test. Similar relationships were also observed for the other deformation temperatures analyzed.

Based on an analysis of the numerical simulation results, it was found that although the global thermomechanical parameters differ between torsion and the extrusion process, the local thermomechanical conditions in the representative regions used for microstructural characterization and microhardness measurements are similar in both analyzed processes.

### 3.2. Results and Microstructural Analysis—Light Microscopy

**Figure 14:**
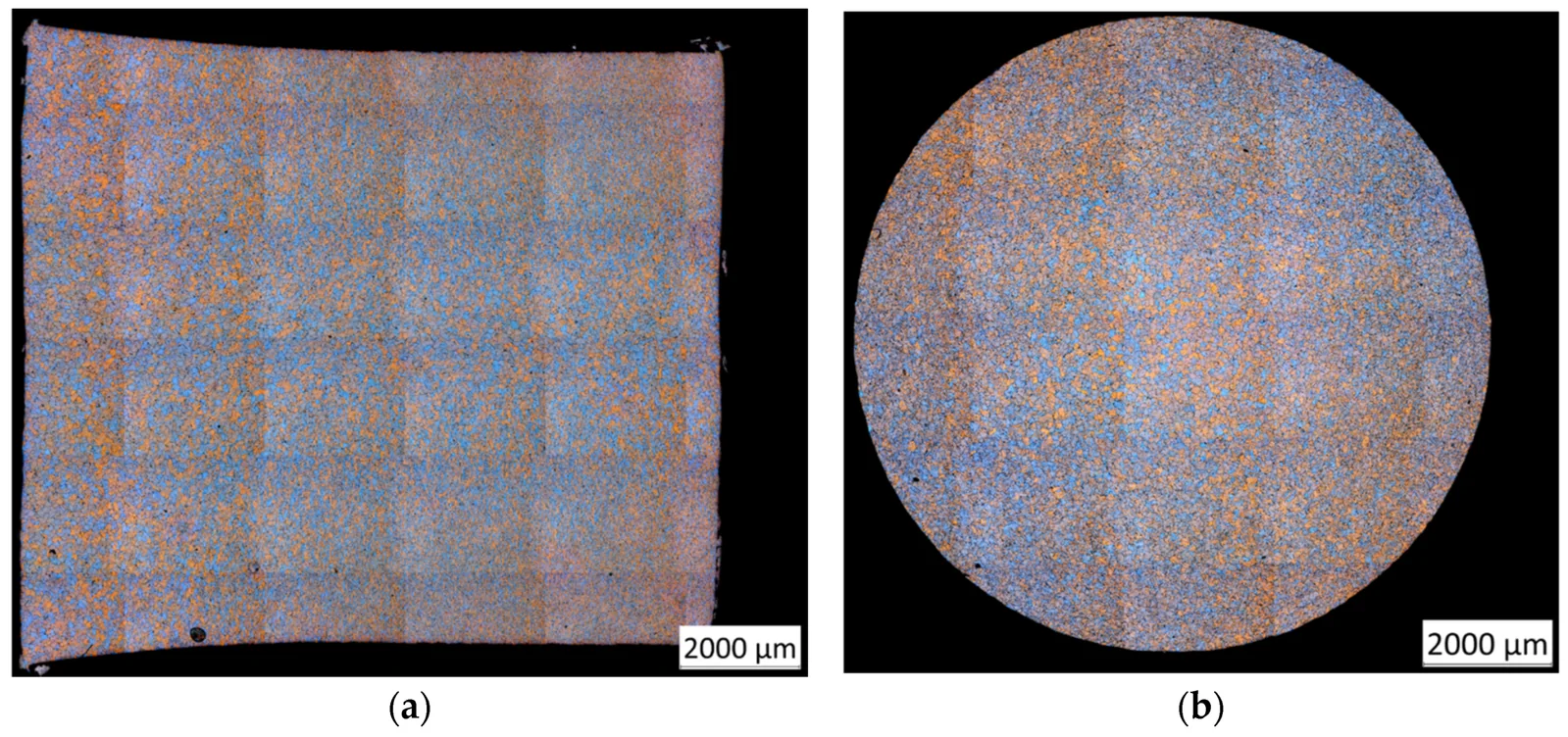
Example microstructures of EN AW-6060 aluminum alloy specimens after physical modeling of the extrusion process using a torsion test: deformation temperature—400 °C, cooling rate—1.7 °C/s. (**a**) Longitudinal section, (**b**) transverse section.

Specimens obtained from both the extrusion and torsion processes underwent standard metallographic preparation, including grain structure revelation using the Barker etching method. The microstructure was then examined using optical microscopy. After analyzing both cross-sections and longitudinal sections, the authors decided to focus the remainder of the study on longitudinal sections, as these proved to be more representative. Figure 14 shows an example comparison of transverse and longitudinal sections for a specimen subjected to torsion at 400 °C and cooled at a rate of 1.7 °C/s.

An analysis of the microstructure images shown in Figure 14 reveals changes in the grain size of the alloy under study. The largest grains were found in the center of the specimens, where the strain values were lowest, while the smallest grains were observed in the near-surface regions, where the strain values were highest. Microstructural analysis showed that the specimen’s texture is more clearly visible in longitudinal sections. A similar trend, albeit less pronounced, was also observed in specimens obtained through the extrusion process under semi-industrial conditions.

**Figure 15:**
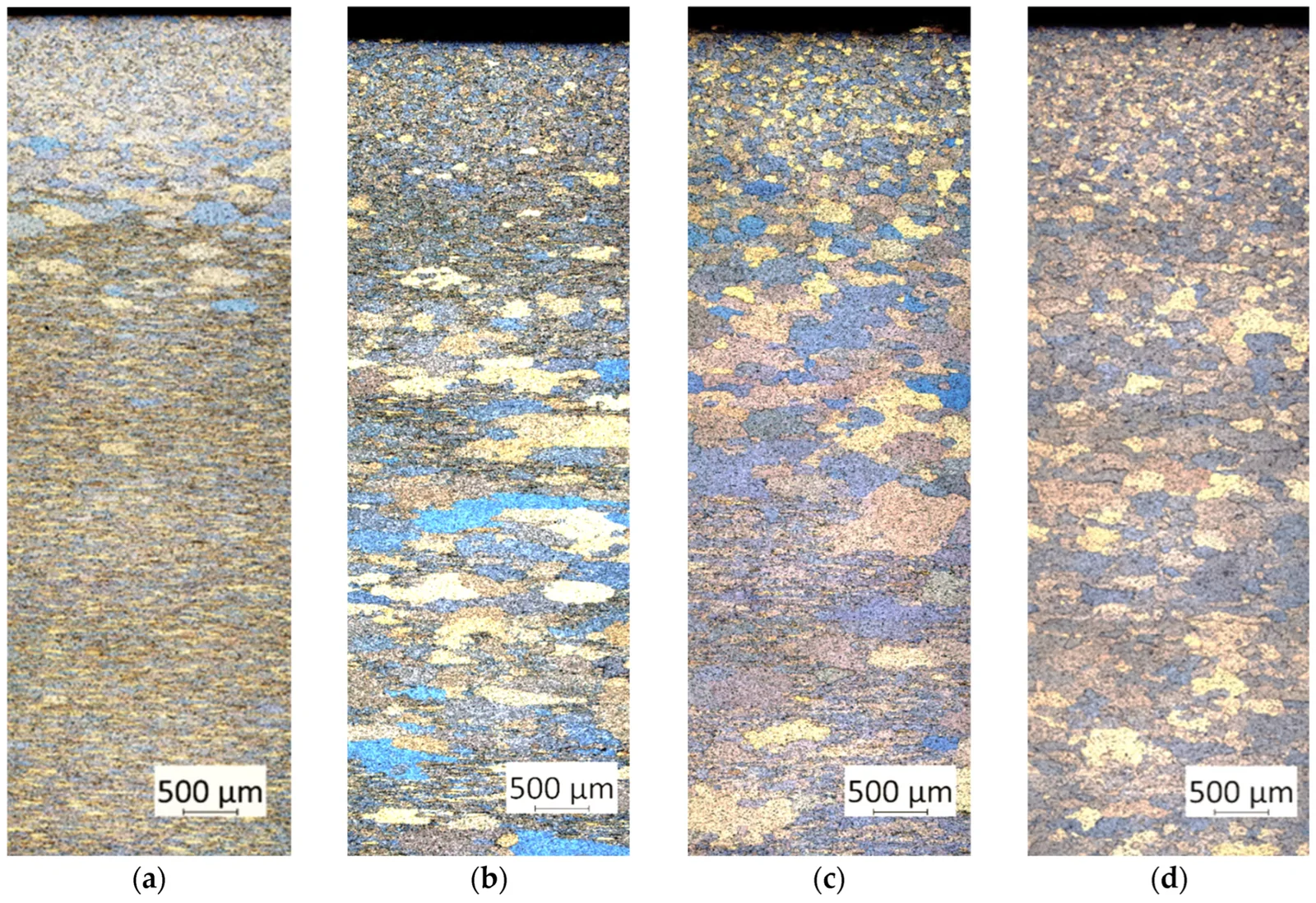
Microstructure of specimens obtained after the extrusion process with forced air cooling—cooling rate of 1.7 °C/s, longitudinal cross-sections: (**a**) 400 °C, (**b**) 450 °C, (**c**) 500 °C, (**d**) 550 °C.

**Figure 16:**
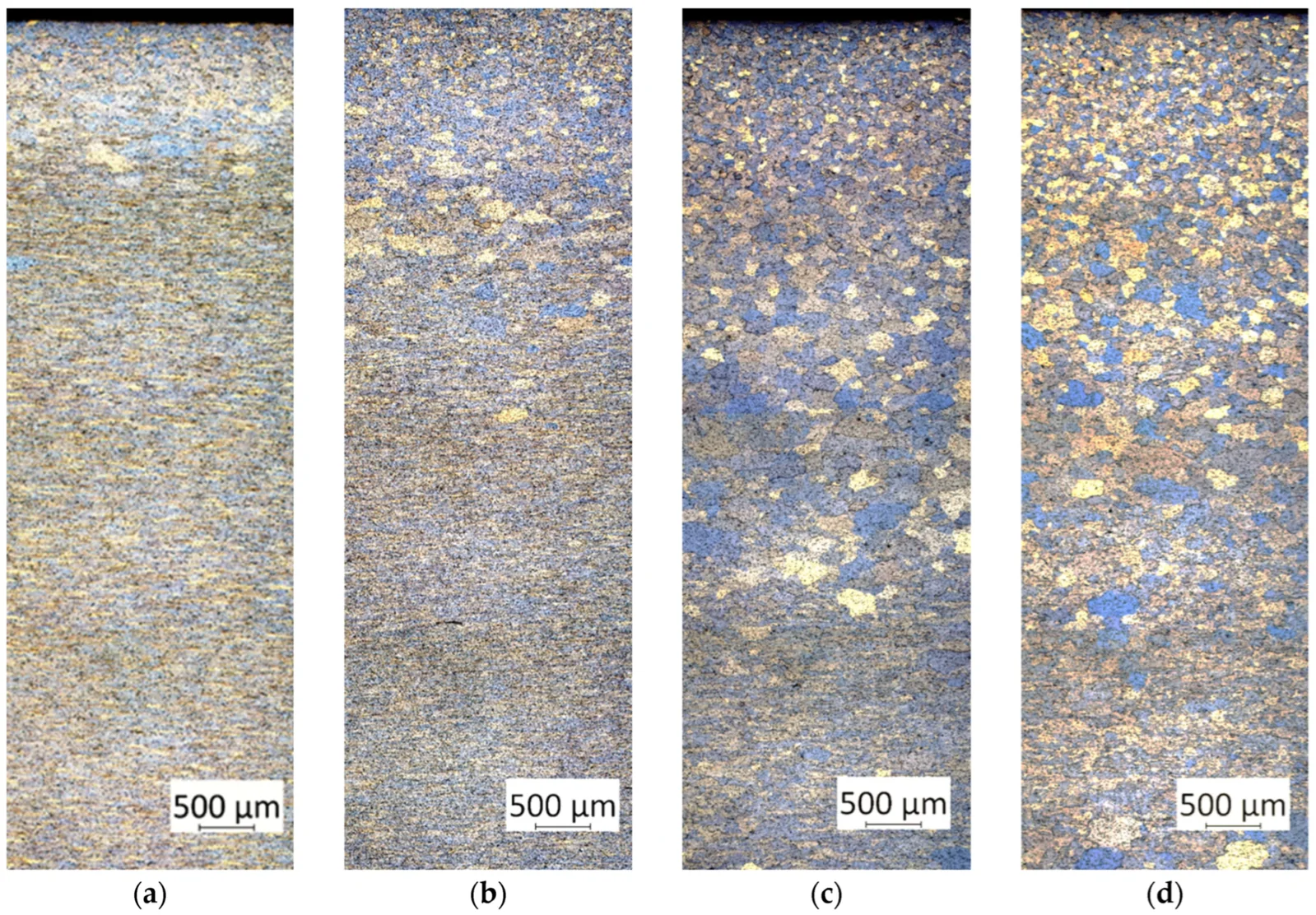
Microstructure of specimens obtained after extrusion with water mist cooling—cooling rate of 15 °C/s, longitudinal sections: (**a**) 400 °C, (**b**) 450 °C, (**c**) 500 °C, (**d**) 550 °C.

**Figure 17:**
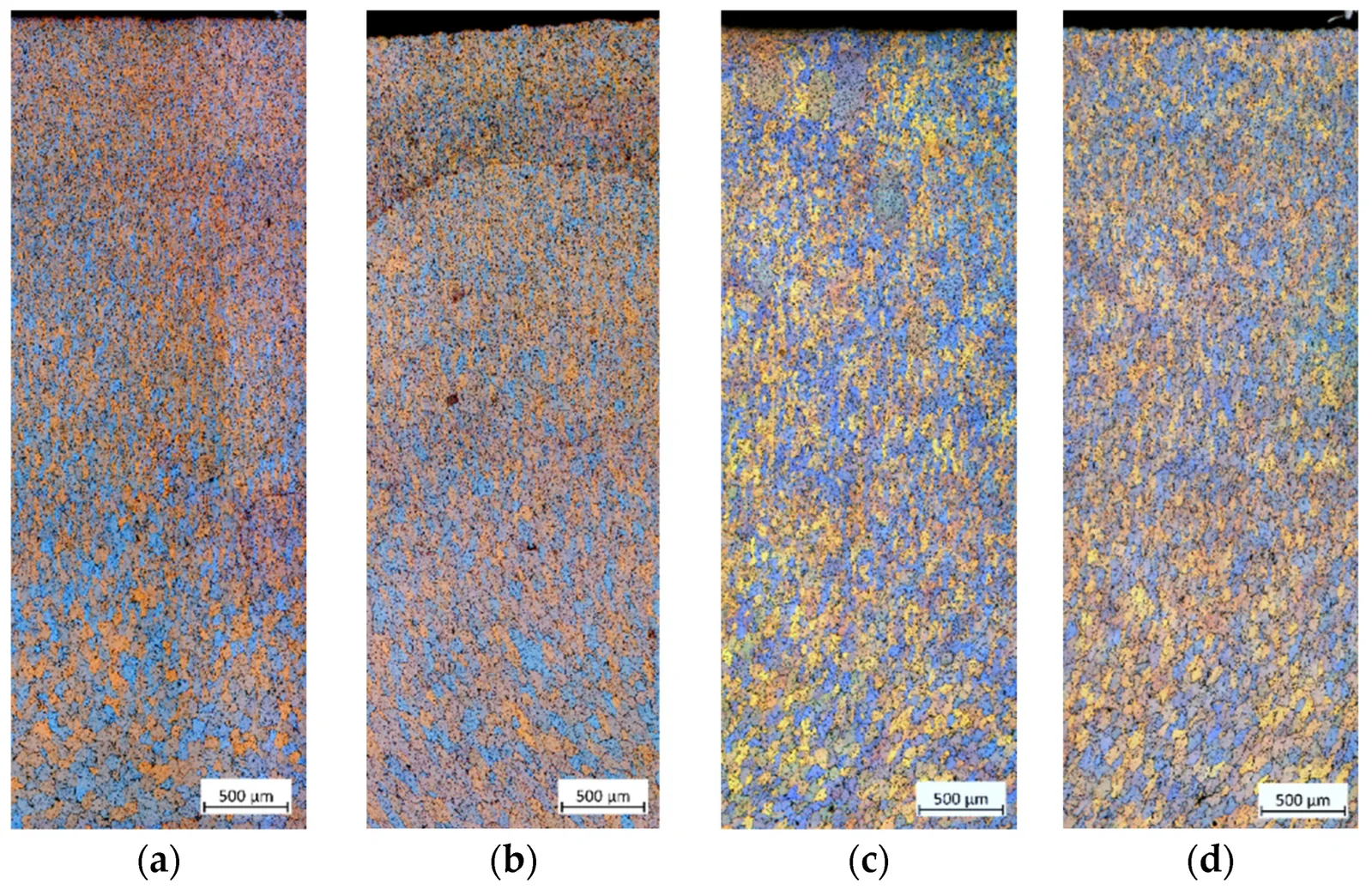
Microstructure of specimens obtained after the torsion process—cooling rate of 1.7 °C/s, longitudinal cross-sections: (**a**) 400 °C, (**b**) 450 °C, (**c**) 500 °C, (**d**) 550 °C.

**Figure 18:**
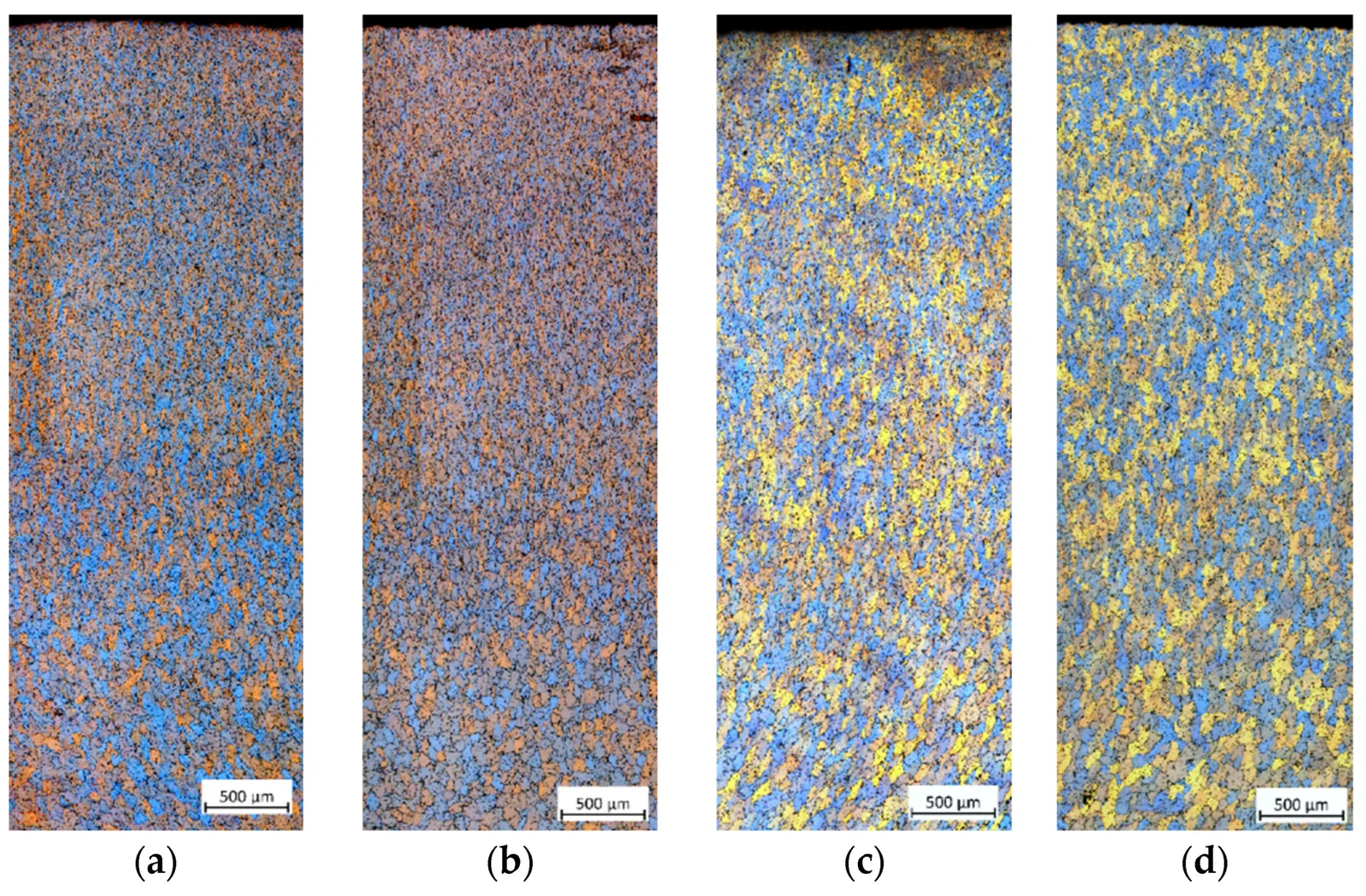
Microstructure of specimens obtained after the torsion process—cooling rate of 15 °C/s, longitudinal sections: (**a**) 400 °C, (**b**) 450 °C, (**c**) 500 °C, (**d**) 550 °C.

**Figure 19:**
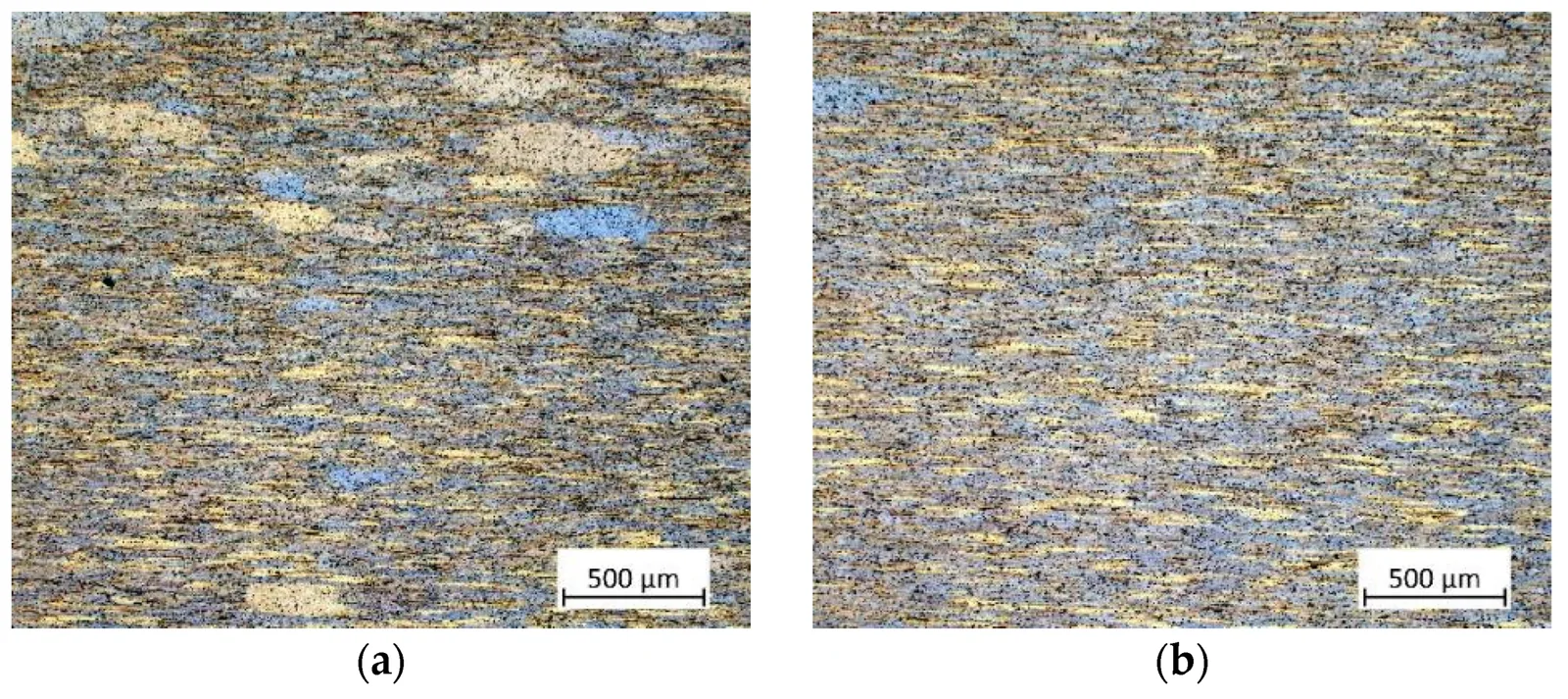
Comparison of the microstructure of specimens after the semi-industrial extrusion process—0.6 of the radius, longitudinal cross-section, extrusion temperature of 400 °C: (**a**) forced-air cooling—cooling rate of 1.7 °C/s; (**b**) water mist cooling—cooling rate of 15 °C/s.

**Figure 20:**
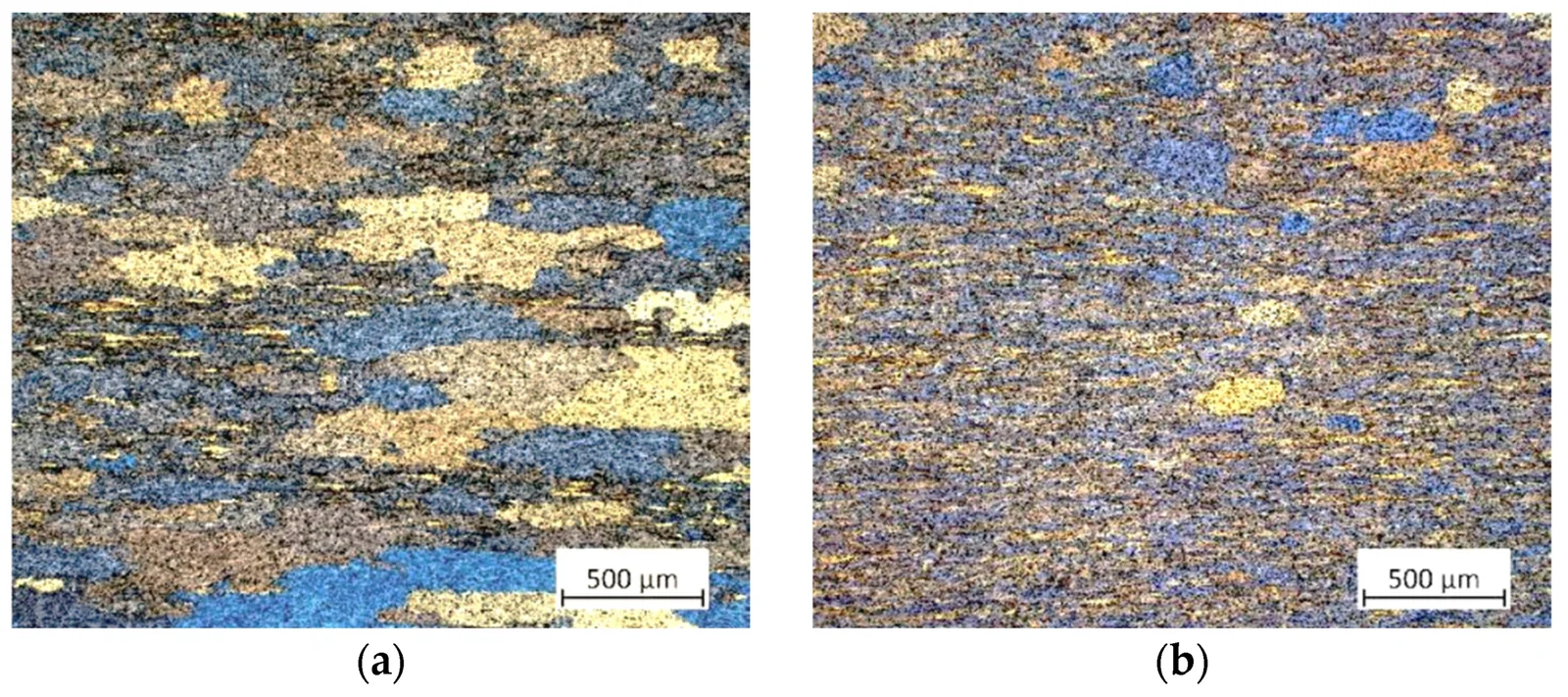
Comparison of the microstructure of specimens after the semi-industrial extrusion process—0.6-radius region, longitudinal cross-section, extrusion temperature of 450 °C: (**a**) forced-air cooling—cooling rate of 1.7 °C/s; (**b**) water mist cooling—cooling rate of 15 °C/s.

**Figure 21:**
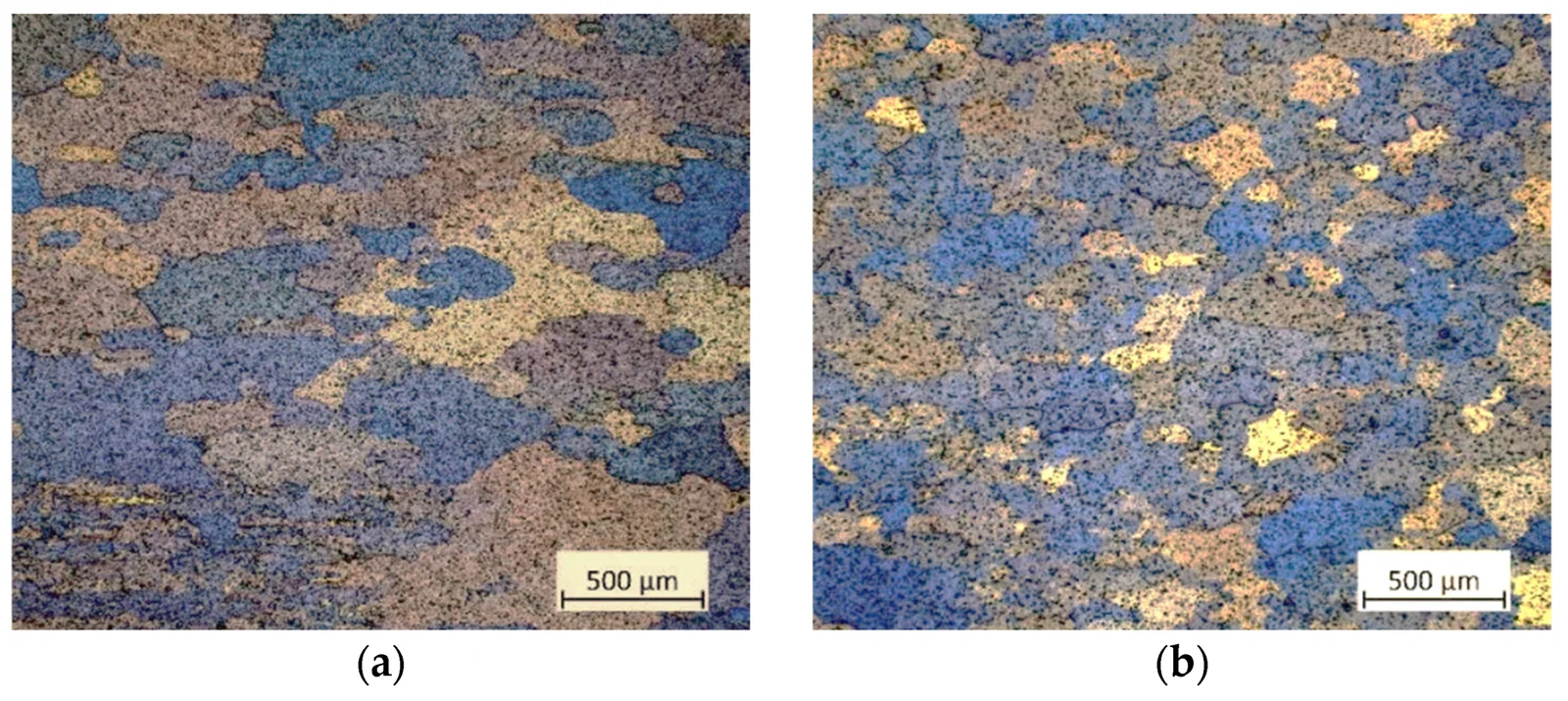
Comparison of the microstructure of specimens after the semi-industrial extrusion process—0.6-radius region, longitudinal cross-section, extrusion temperature of 500 °C: (**a**) forced air cooling—cooling rate of 1.7 °C/s; (**b**) water mist cooling—cooling rate of 15 °C/s.

**Figure 22:**
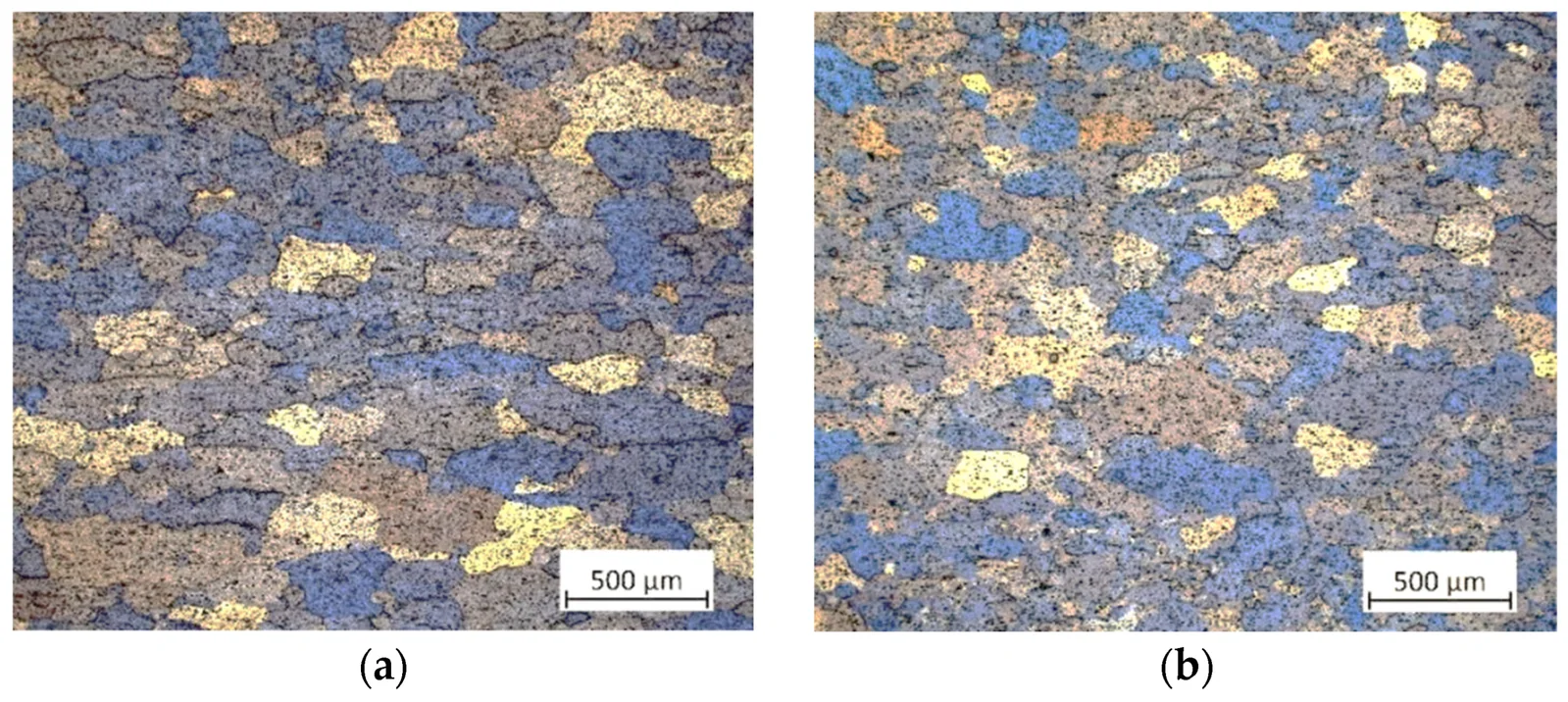
Comparison of the microstructure of specimens after the semi-industrial extrusion process—0.6-radius region, longitudinal cross-section, extrusion temperature of 550 °C: (**a**) forced-air cooling—cooling rate of 1.7 °C/s; (**b**) water mist cooling—cooling rate of 15 °C/s.

**Figure 23:**
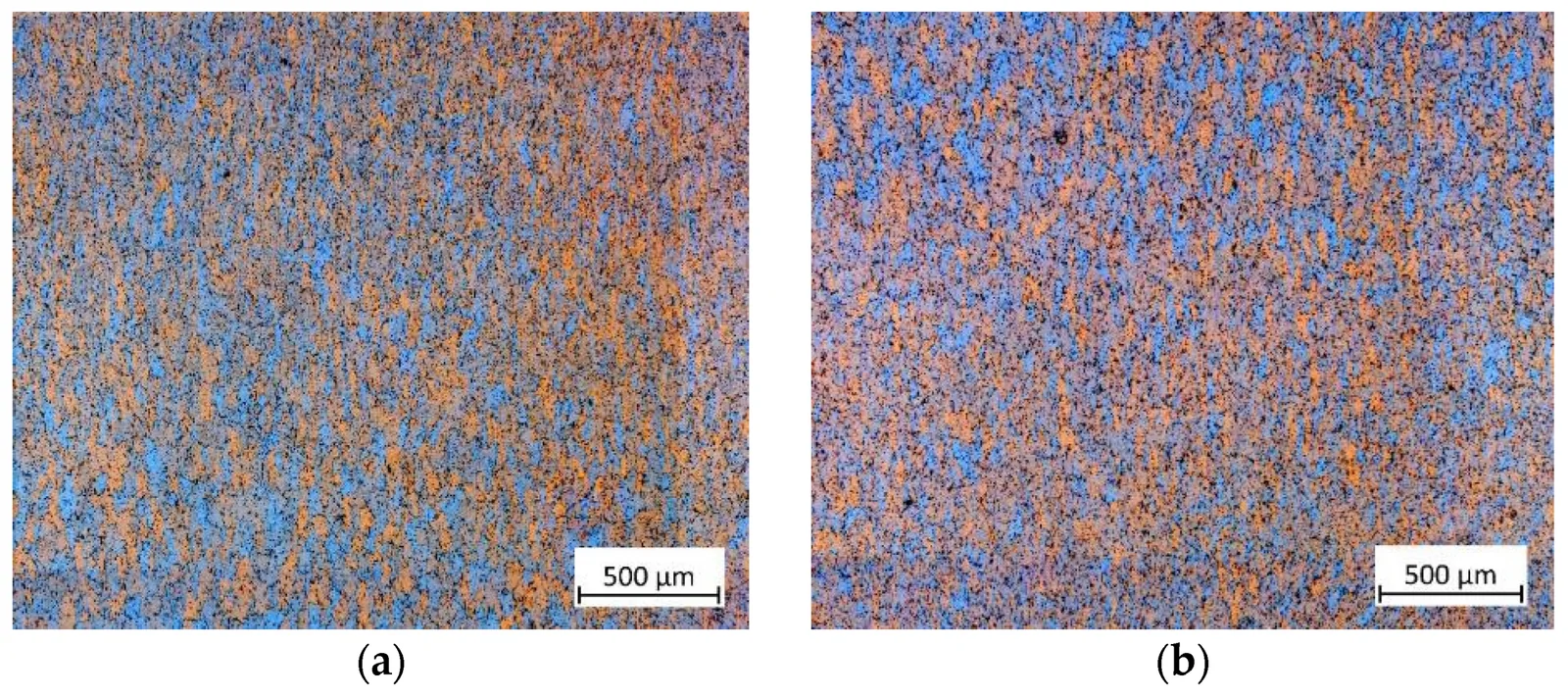
Comparison of the microstructure of specimens after the torsion process—0.6-radius region, longitudinal cross-section, torsion temperature of 400 °C: (**a**)—cooling rate of 1.7 °C/s; (**b**)—cooling rate of 15 °C/s.

**Figure 24:**
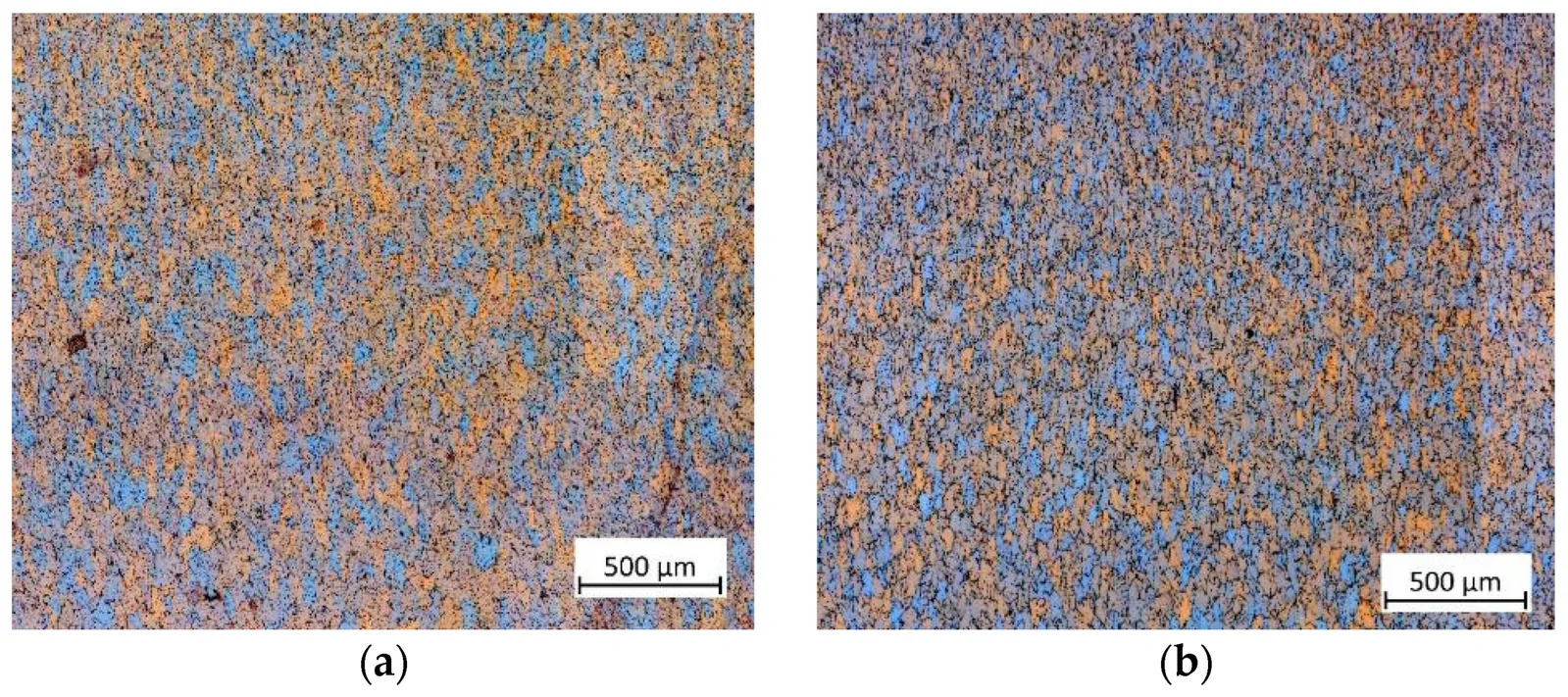
Comparison of the microstructure of specimens after the torsion process—0.6-radius area, longitudinal section, torsion temperature of 450 °C: (**a**)—cooling rate of 1.7 °C/s; (**b**)—cooling rate of 15 °C/s.

**Figure 25:**
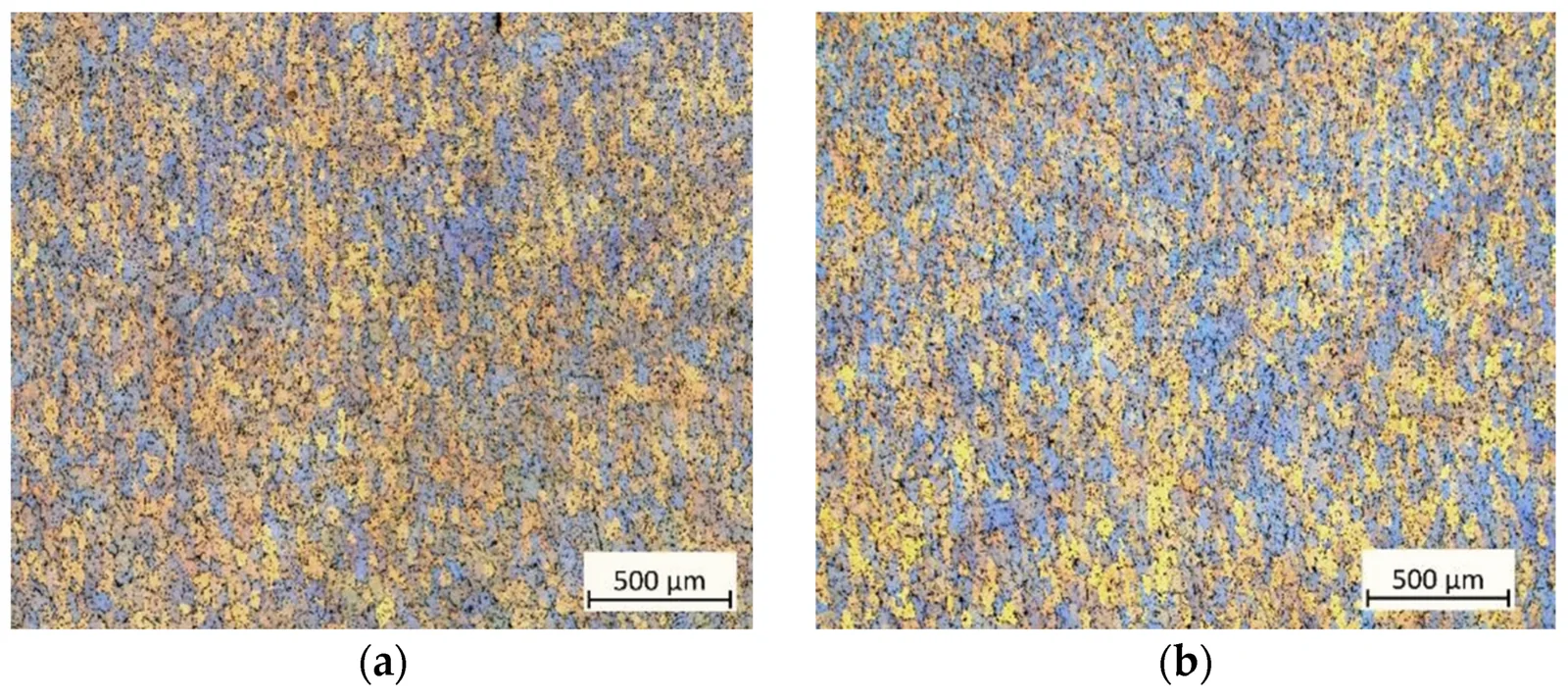
Comparison of the microstructure of specimens after the torsion process—0.6-radius area, longitudinal section, torsion temperature of 500 °C: (**a**)—cooling rate of 1.7 °C/s; (**b**)—cooling rate of 15 °C/s.

**Figure 26:**
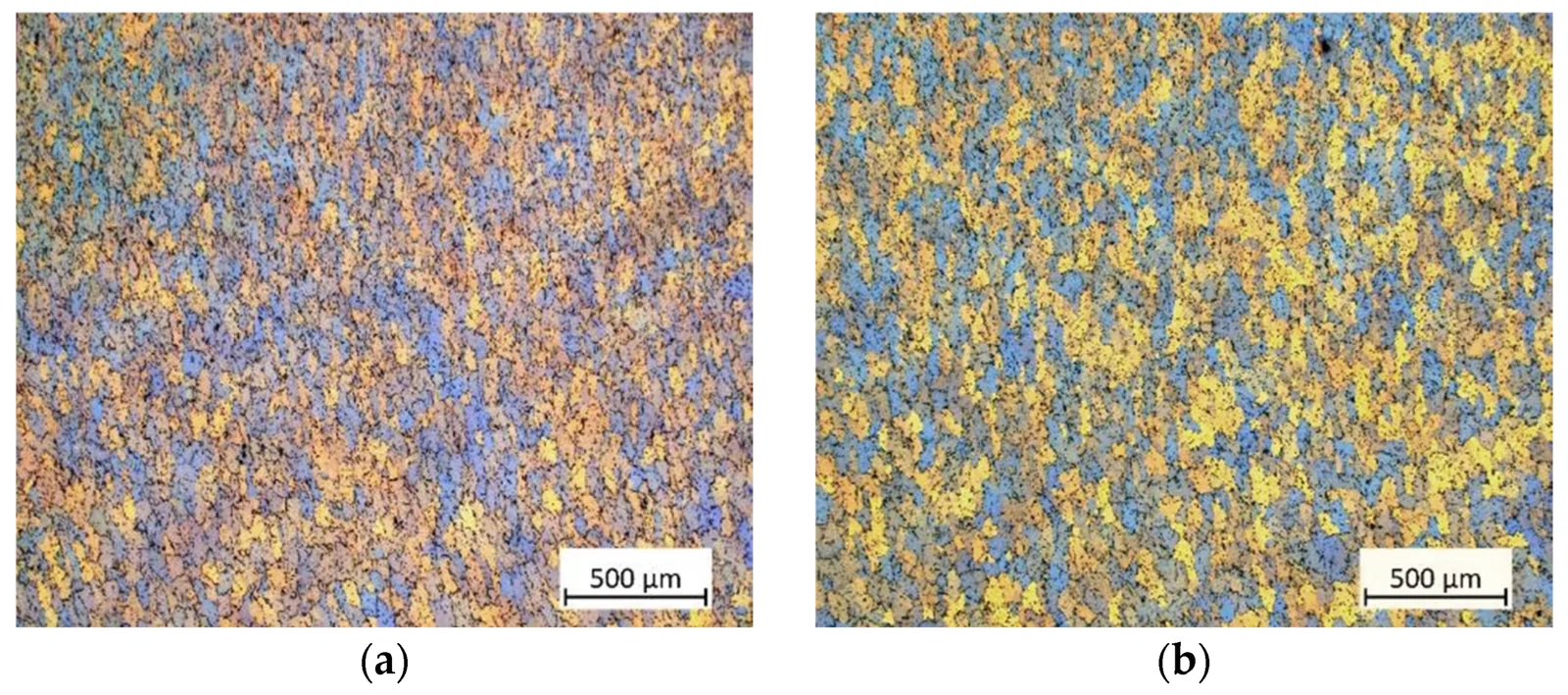
Comparison of the microstructure of specimens after the torsion process—0.6-radius area, longitudinal section, torsion temperature of 550 °C: (**a**)—cooling rate of 1.7 °C/s; (**b**)—cooling rate of 15 °C/s.

Figure 15, Figure 16, Figure 17, Figure 18, Figure 19, Figure 20, Figure 21, Figure 22, Figure 23, Figure 24, Figure 25 and Figure 26 present the results of metallographic analyses of longitudinal cross-sections of the tested material after torsion tests and the corresponding specimens obtained after the extrusion process. The images include results for all tested thermoplastic processing temperatures (400, 450, 500, and 550 °C) and for both cooling rates used: 1.7 and 15 °C/s. Figure 15, Figure 16, Figure 17 and Figure 18 show longitudinal cross-sections through the centerline of the specimen; due to the axial symmetry of the analyzed specimens, only half of the cross-section is shown. For the specimens subjected to the torsion test, specimens were taken from the center of the working length of the specimens. Figure 19, Figure 20, Figure 21, Figure 22, Figure 23, Figure 24, Figure 25 and Figure 26 present the results obtained for the area located at approximately 0.6r from the axis of the tested specimens. This area was selected based on FEM analysis as the most representative for a direct comparison of the two processes, corresponding to areas characterized by similar levels of strain parameters.

Based on the metallographic analysis conducted using light microscopy, it was found that in the microstructure of all analyzed specimens, there was a distinct difference between the specimen axis and the near-surface region. This effect was directly related to the material’s flow characteristics during plastic deformation. The phenomenon was more pronounced in specimens subjected to physical modeling in a torsion test, where the level of plastic deformation increased with increasing distance from the specimen axis.

In the case of microstructural analysis of specimens following the semi-industrial extrusion process, the observed differences resulted from non-uniform material flow in the deformation zone, including the presence of dead zones and areas located near the die contact surface. This led to an increase in the level of plastic deformation in the near-surface zone. Additionally, during the extrusion process, local temperature increases caused by friction occurred, which could have led to the formation of a characteristic coarse-grained microstructure directly on the outer surface.

When analyzing the microstructure of EN AW-6060 alloy specimens in terms of thermoplastic processing temperature, a distinct increase in grain size was observed as the deformation temperature rose, particularly in areas characterized by the highest levels of plastic deformation. This behavior can be attributed to recrystallization processes occurring at elevated temperatures, further promoted by the deformation energy accumulated in the material during plastic deformation.

Regarding the effect of cooling rate after the deformation process, it was observed that at higher thermoplastic processing temperatures (500 and 550 °C), increased cooling intensity partially limited grain growth, effectively “freezing” the microstructure to some extent after deformation. However, the cooling rates used were not high enough to completely inhibit grain growth.

An analysis conducted using light microscopy showed that the observed changes in microstructure morphology are consistent with the results of numerical modeling. In particular, the location of the areas with the highest degree of grain refinement and the direction of microstructural changes corresponded to the distributions of true strain determined by the finite element method. It should be emphasized that the objective of this preliminary study was not to conduct a detailed quantitative analysis of grain size, but rather to assess the potential of the torsion test for characterizing the microstructural evolution occurring during the extrusion process. For this reason, the analysis using light microscopy focused primarily on comparing grain morphology and the nature of microstructural changes in representative areas of both processes. To quantitatively verify the degree of recrystallization for selected, representative processing variants, an additional EBSD analysis was conducted, the results of which are presented later in this paper.

### 3.3. Results and Microstructural Analysis—EBSD Microscopy

This chapter presents the results of the microstructural analysis of the EN AW-6060 alloy after the deformation process, using Electron Backscatter Diffraction (EBSD) analysis. Due to the representative nature of the analyses and the large number of variants examined, the results for selected specimens deformed at temperatures of 450 and 550 °C are presented below.

**Figure 27:**
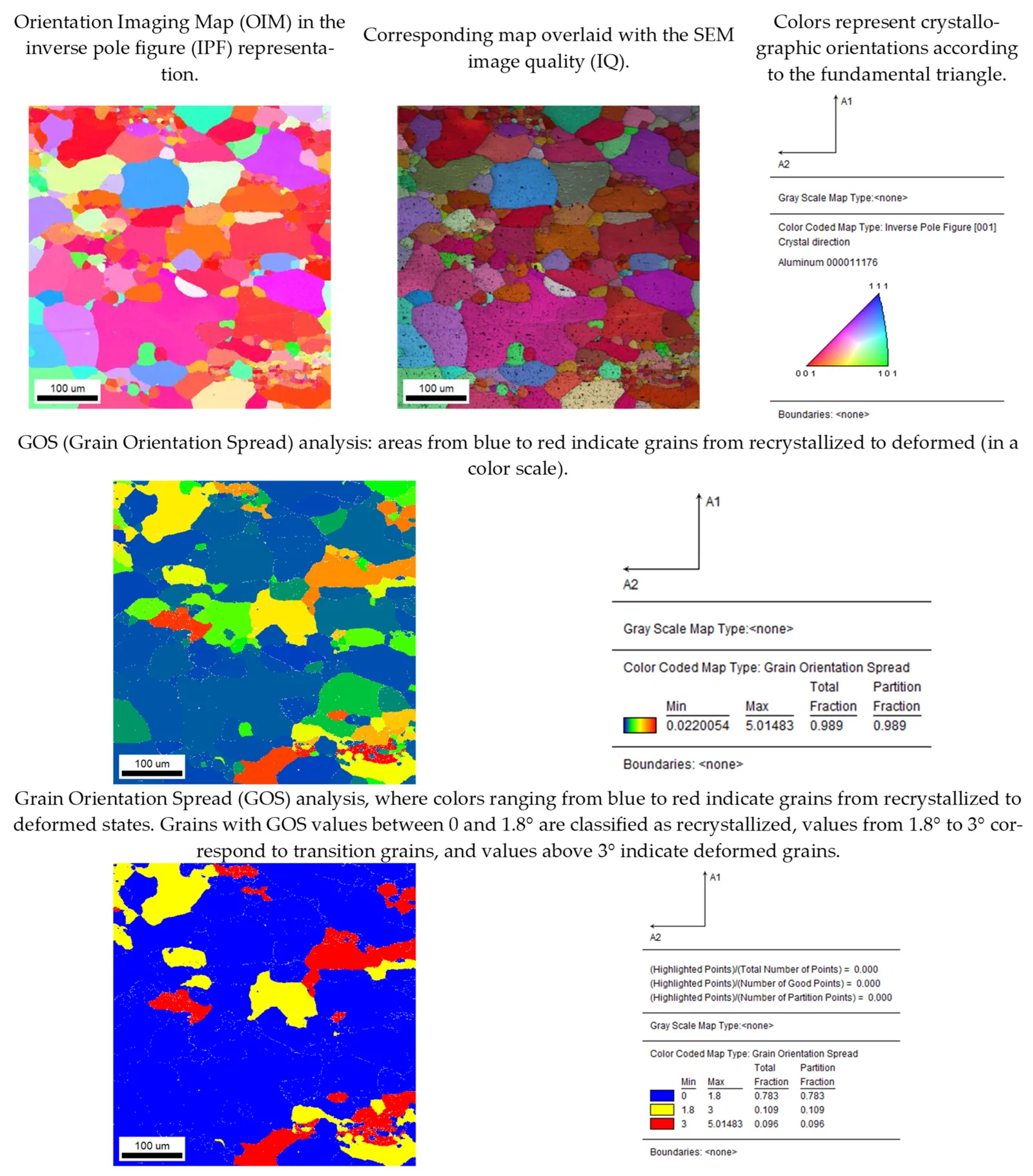
Results of EBSD analysis of EN AW-6060 aluminum alloy specimens after a semi-industrial extrusion process at 450 °C, cooled with water mist—cooling rate of 15 °C/s.

**Figure 28:**
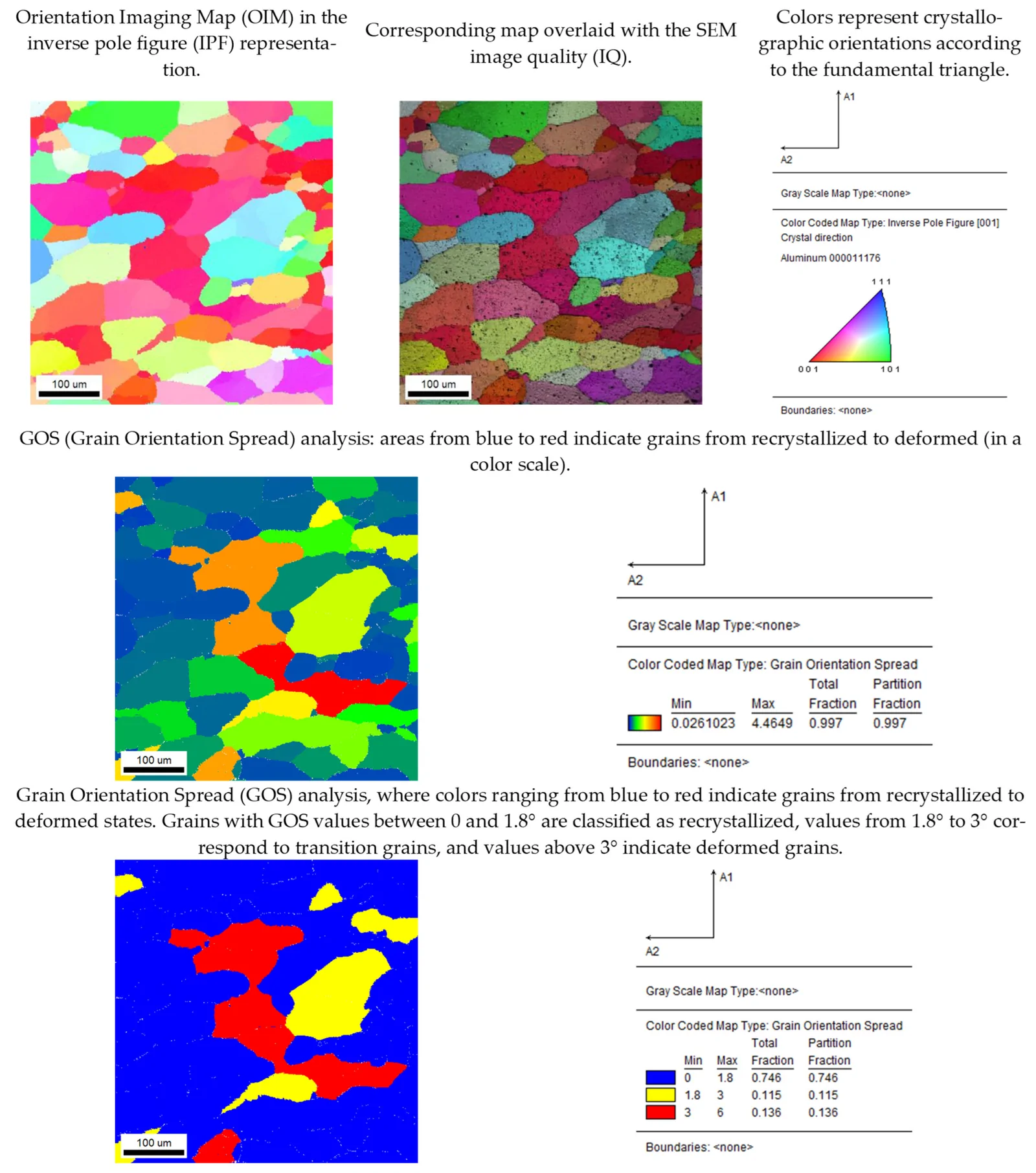
Results of EBSD analysis of EN AW-6060 aluminum alloy specimens after a semi-industrial extrusion process at 550 °C, cooled with water mist—cooling rate of 15 °C/s.

**Figure 29:**
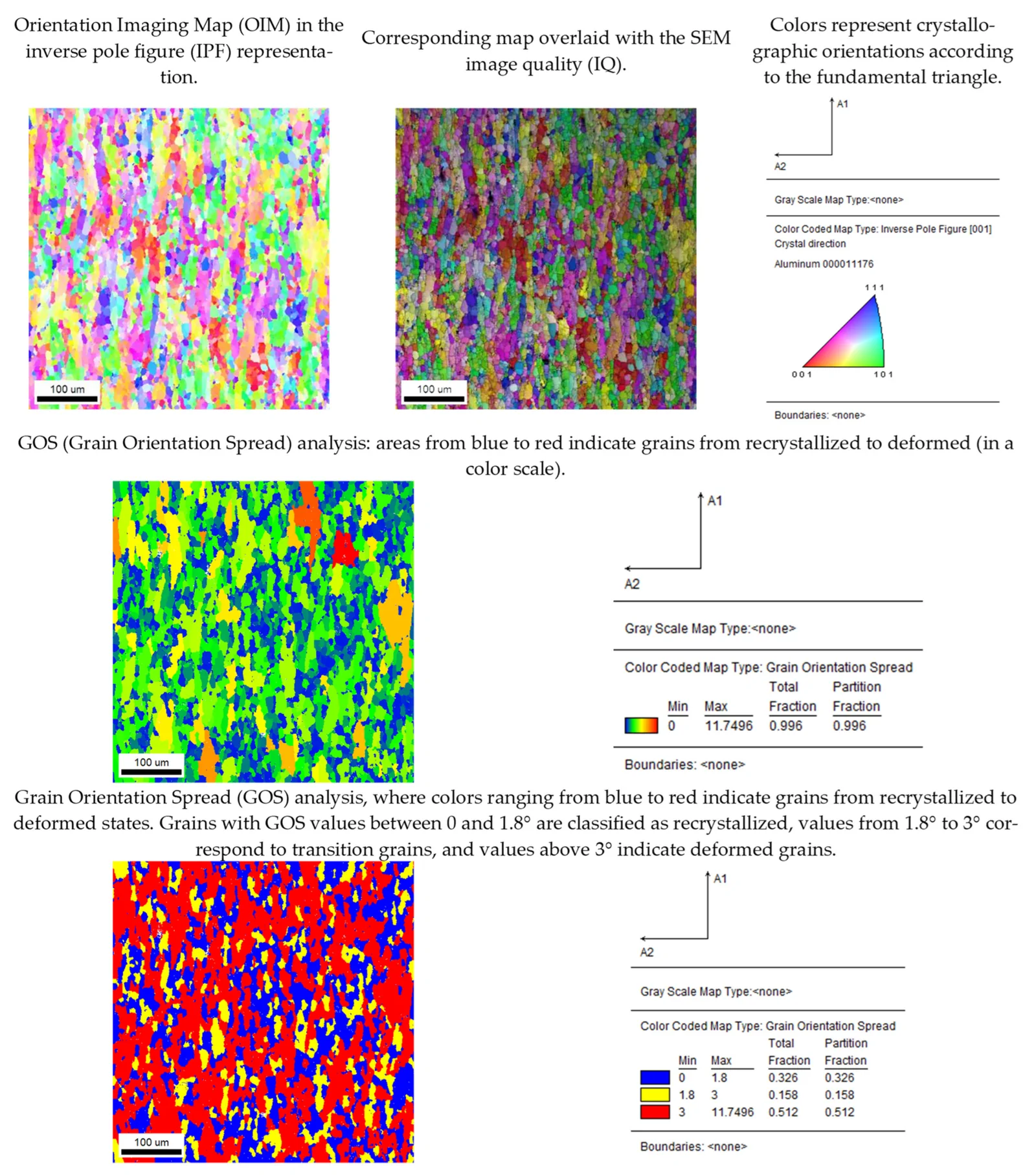
Results of EBSD analysis of EN AW-6060 aluminum alloy specimens after physical modeling at 450 °C using a torsion plastometer—cooling rate of 15 °C/s.

**Figure 30:**
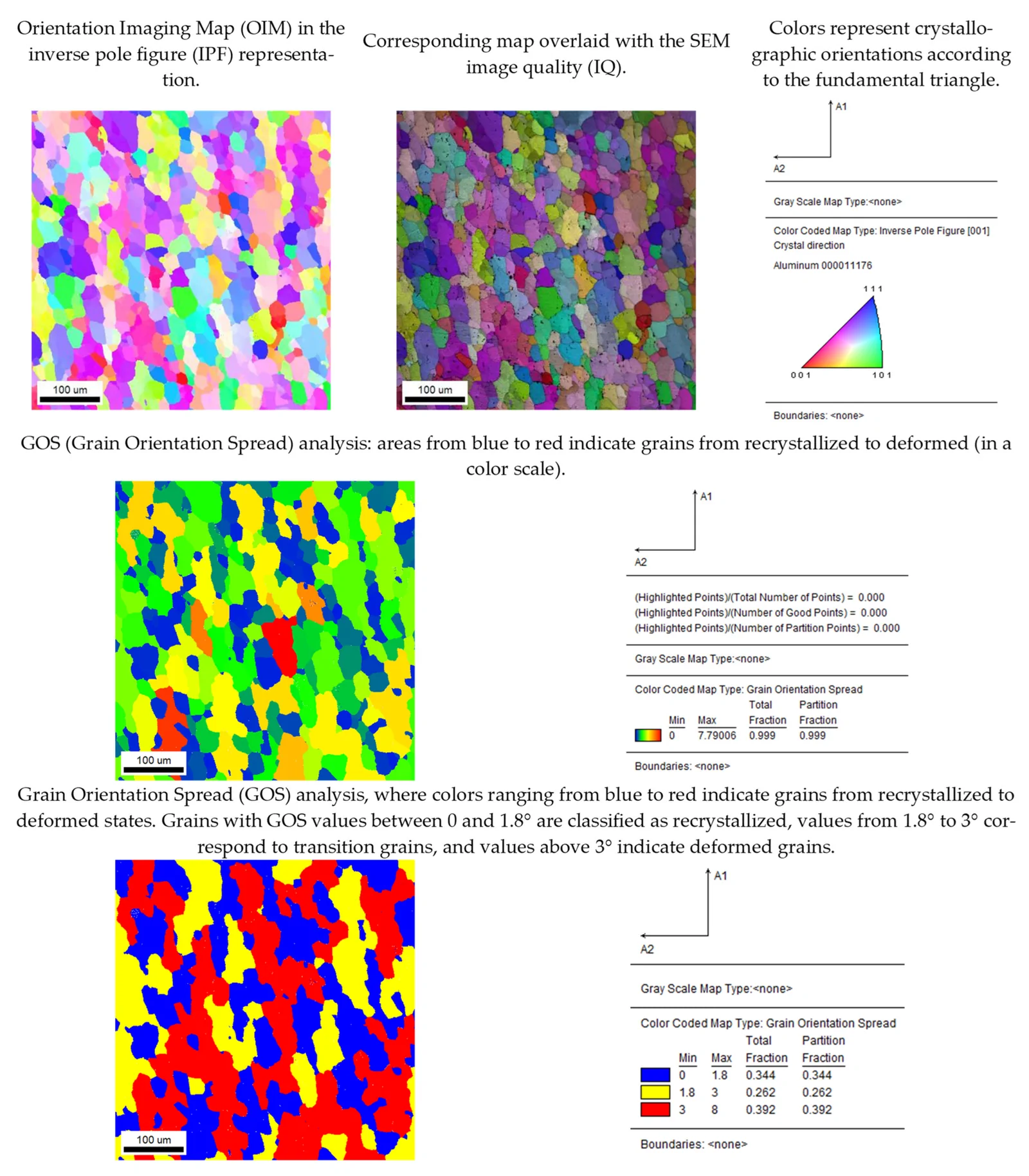
Results of EBSD analysis of EN AW-6060 aluminum alloy specimens after physical modeling at 550 °C using a torsion plastometer—cooling rate of 15 °C/s.

All specimens were oriented in the same manner, according to a predetermined scheme. The specimens were taken from areas characterized by a similar degree of deformation (longitudinal cross-section at approximately 0.6r from the specimen axis), in accordance with the arrangement shown in Figure 8. Representative specimens deformed at 450 and 550 °C were selected for detailed quantitative EBSD analysis because these conditions are frequently investigated in the authors’ research and in studies conducted in cooperation with industrial partners for the investigated alloy and process. The selection of these two temperatures allows microstructural evolution in hot torsion and extrusion to be compared at two representative temperature levels. The quantitative EBSD analysis complements the metallographic observations performed over the full investigated temperature range. A cooling rate of 15 °C/s (approximately 17 °C/s for the extrusion process) was selected to visualize the crystallographic texture and the degree of recrystallization “frozen” immediately after the deformation process. Scanning was performed over an area measuring 500 × 500 µm with a step size of 0.4 µm. One representative EBSD measurement area was analyzed for each investigated condition. Grain boundaries were identified using a misorientation threshold of 15°, and the minimum grain size considered in the analysis was 0.8 µm. Non-indexed points were removed during data cleaning prior to quantitative analysis. Neighbor Orientation Correlation was applied during the data cleaning procedure. Specimen results are shown in Figure 27, Figure 28, Figure 29 and Figure 30.

Metallographic examinations using Electron Backscatter Diffraction (EBSD) analysis revealed a significant increase in grain size in specimens subjected to semi-industrial extrusion at a higher temperature (550 °C). Specimens extruded at 450 °C exhibited a fine-grained microstructure. In the specimens extruded at 500 °C, despite the use of a cooling rate of 15 °C/s, a mixed structure was also observed: some grains were recrystallized and relatively large, while the others were fine. The fine grains were located mainly along the deformation bands formed during the extrusion process.

In specimens subjected to physical modeling using a torsion plastometer, the deformation mechanism proceeded somewhat differently. The deformation bands were arranged along the direction of the torsion process at an angle to the orientation of the specimen. The observed bands correspond to the characteristic shear bands typical of the torsion process. The differences between specimens deformed at 450 °C and 500 °C were primarily related to grain size and the degree of recrystallization. In the specimens deformed at 550 °C, a slightly higher proportion of recrystallized and deformed regions was observed compared to the specimens deformed at 450 °C.

**Figure 31:**
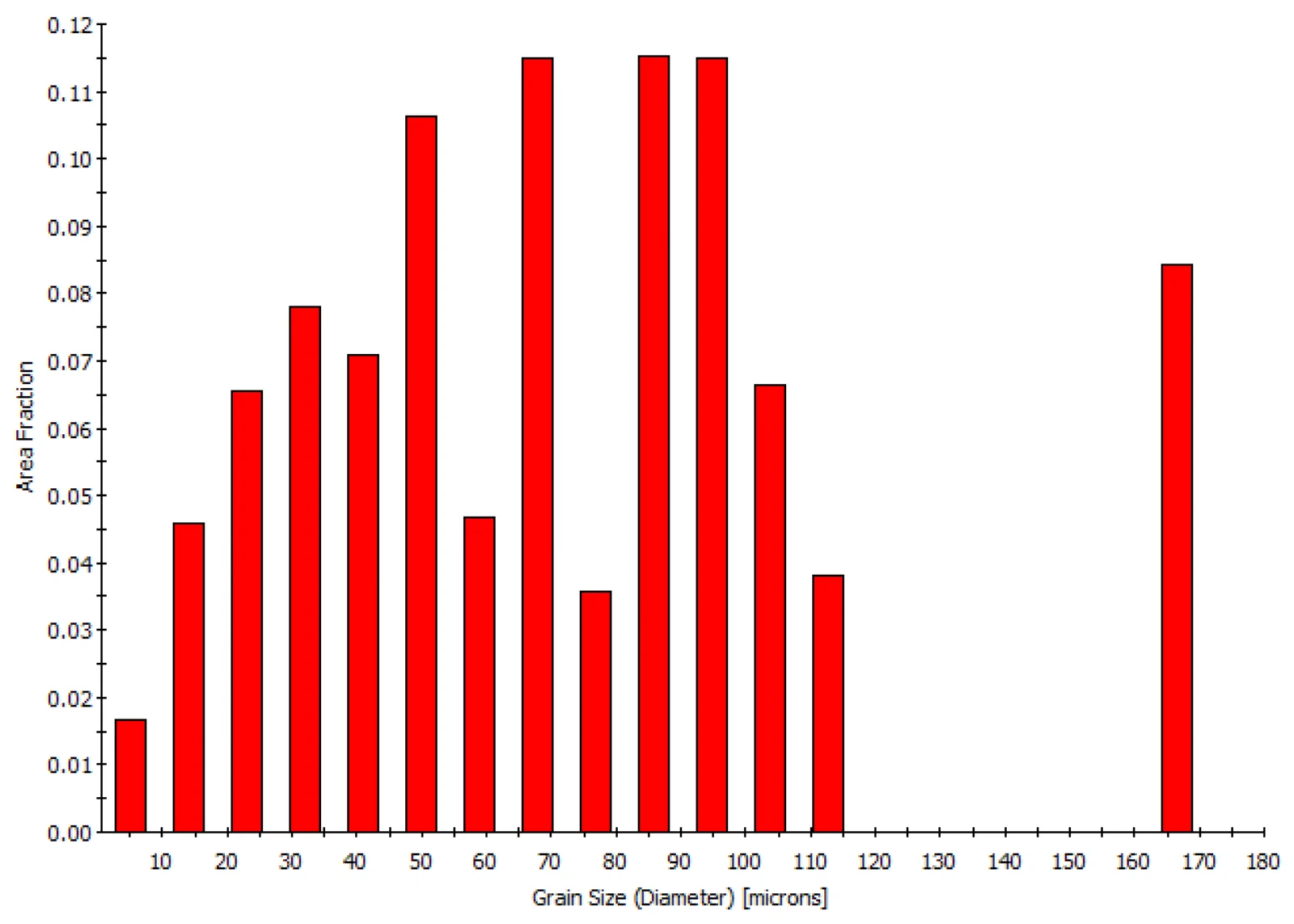
Grain-size distribution obtained from EBSD analysis for the specimen subjected to extrusion at 450 °C.

**Figure 32:**
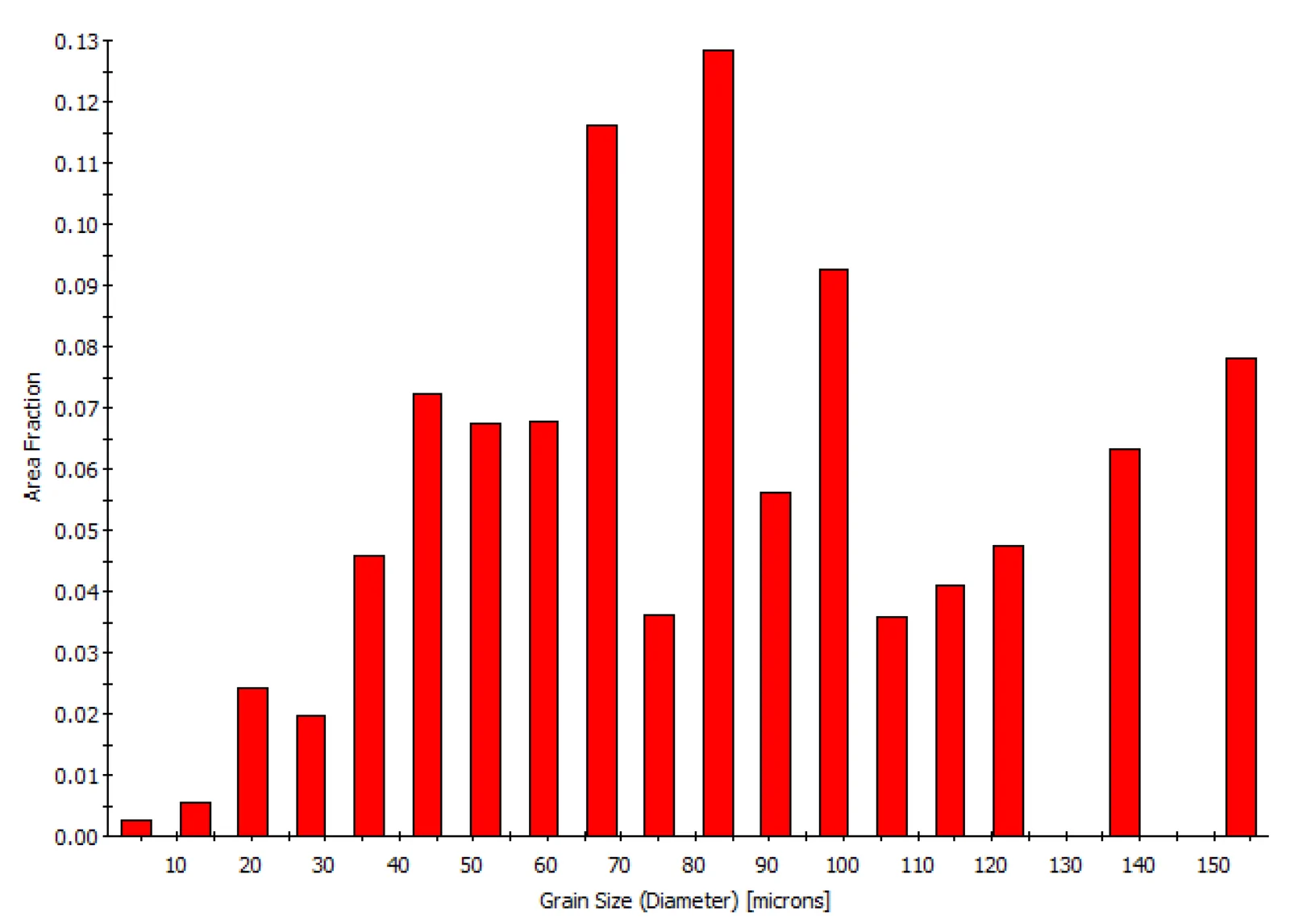
Grain-size distribution obtained from EBSD analysis for the specimen subjected to extrusion at 550 °C.

**Figure 33:**
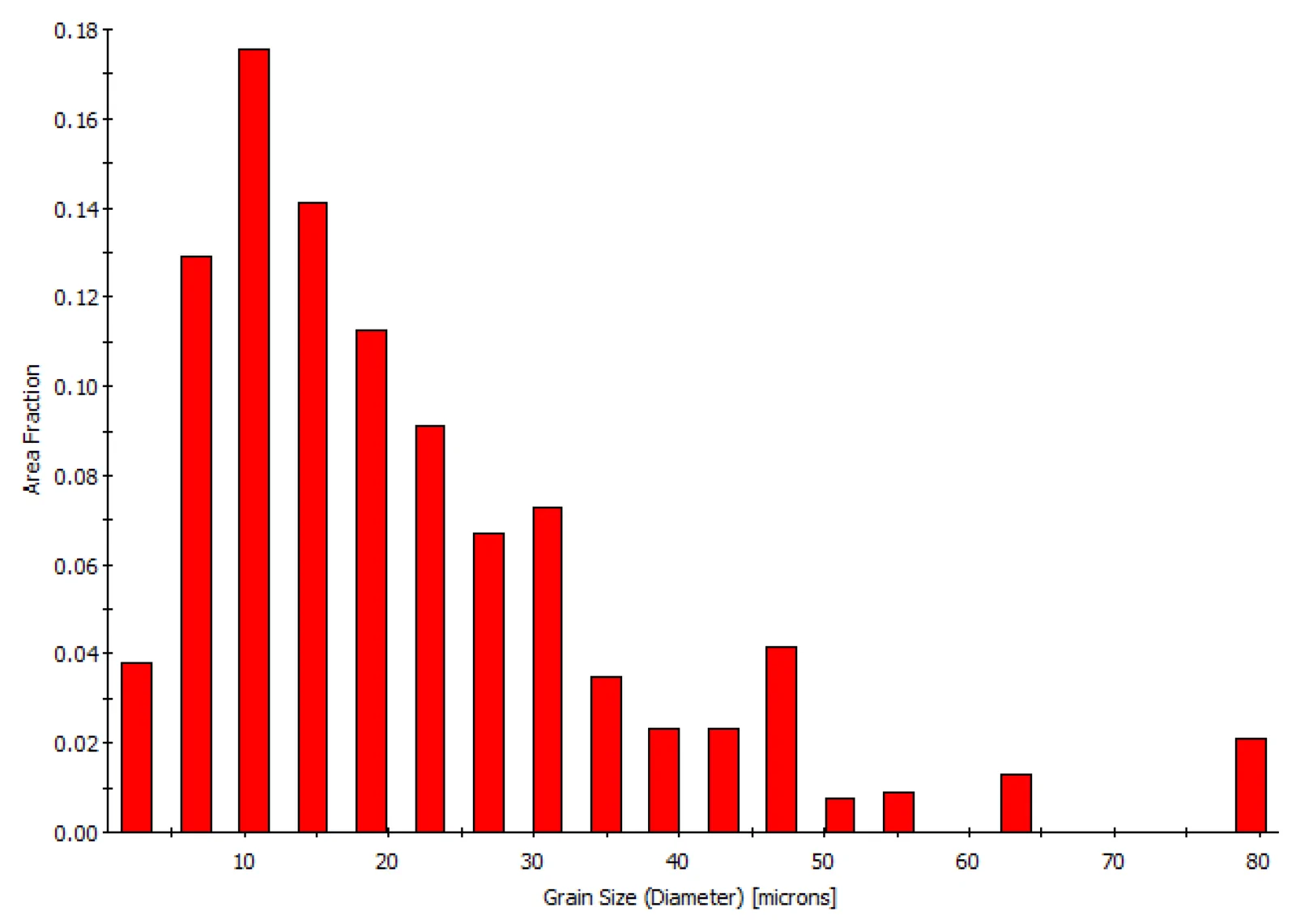
Grain-size distribution obtained from EBSD analysis for the specimen subjected to torsion at 450 °C.

**Figure 34:**
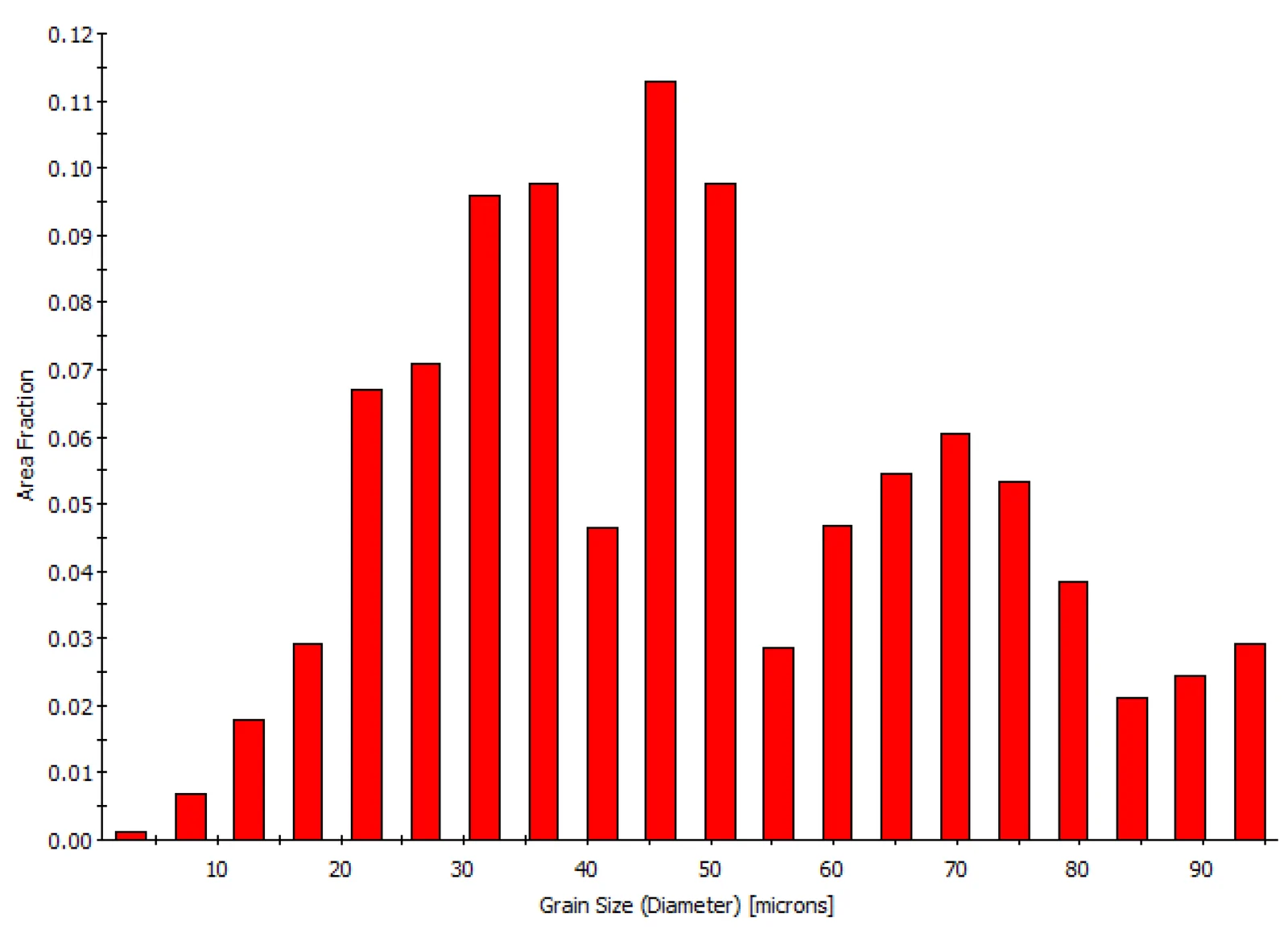
Grain-size distribution obtained from EBSD analysis for the specimen subjected to torsion at 550 °C.

Based on the above EBSD results, a grain-size analysis was performed for four representative conditions: extrusion at 450 and 550 °C and torsion at 450 and 550 °C. The analysis included the grain-size distributions obtained from the EBSD data, presented as histograms in Figure 31, Figure 32, Figure 33 and Figure 34, and the corresponding mean grain diameter and standard deviation, summarized in Table 5. The standard deviations reported for grain size represent the dispersion of grain diameters within the analyzed EBSD map and do not represent variability between independent experimental replicates. The results showed clear differences between the two deformation processes. The extruded specimens exhibited larger mean grain diameters, increasing from 72.0 µm at 450 °C to 84.8 µm at 550 °C, whereas the corresponding values for the torsion specimens were 21.6 µm and 48.7 µm, respectively. In both deformation processes, increasing the deformation temperature resulted in an increase in the mean grain diameter.

A quantitative assessment of the degree of recrystallization was performed using the GOS (Grain Orientation Spread) parameter, classifying the grains as recrystallized (GOS < 1.8°), partially recrystallized (1.8° ≤ GOS ≤ 3°), and deformed (GOS > 3°). The selected GOS thresholds were established empirically based on the authors’ previous EBSD analyses of aluminum alloys with similar chemical compositions. In these studies, the GOS values were correlated with experimentally observed microstructural states representing different stages of recrystallization. Based on this comparison, the thresholds were selected to distinguish recrystallized, partially recrystallized, and deformed grain populations and were applied consistently to all datasets analyzed in the present study. In the specimens obtained through the extrusion process, the proportion of recrystallized grains was 78.3% at 450 °C and 74.6% at 550 °C, respectively. At the same time, the proportion of partially recrystallized and deformed grains remained at a similar level, amounting to 10.9% and 9.5%, respectively, for 450 °C, and 11.5% and 13.6% for 550 °C. For specimens subjected to physical modeling, the proportion of recrystallized grains was 32.6% and 34.4%, respectively, while the proportion of partially recrystallized grains increased from 15.8% to 26.2%, accompanied by a decrease in the proportion of deformed grains from 51.2% to 39.2%. The results of the EBSD analysis are consistent with observations made using light microscopy. In both analyzed processes, an increase in the deformation temperature led to distinct changes in microstructure morphology, manifested by an increase in grain size and a change in the proportion of individual grain classes determined by the GOS method. The obtained results confirm that appropriately selected torsion test conditions allow for a qualitative representation of the direction of microstructure evolution observed during the extrusion process.

The pronounced difference in recrystallized fraction between the extrusion and torsion processes, despite the use of comparable local deformation conditions, can be attributed to the fundamentally different deformation paths and process histories. In the present study, the comparison was performed for regions characterized by similar temperature, strain rate and local true strain; however, the stress state and deformation mode were substantially different in the two processes. During extrusion, the material undergoes a complex combination of deformation modes associated with the material flow through the die, whereas torsion is dominated by shear deformation. In addition, the thermal histories of the two processes are not identical. In the torsion tests, the cooling rate after deformation was precisely controlled at an approximately constant rate, whereas the thermal history during extrusion was more complex due to the interaction between deformation, friction and heat transfer. Consequently, matching selected local thermomechanical parameters does not result in identical deformation and thermal histories. These differences can affect the stored deformation energy, dislocation structure and conditions for recrystallization, and therefore may explain the substantially higher recrystallized fraction observed after extrusion than after torsion.

**Figure 35:**
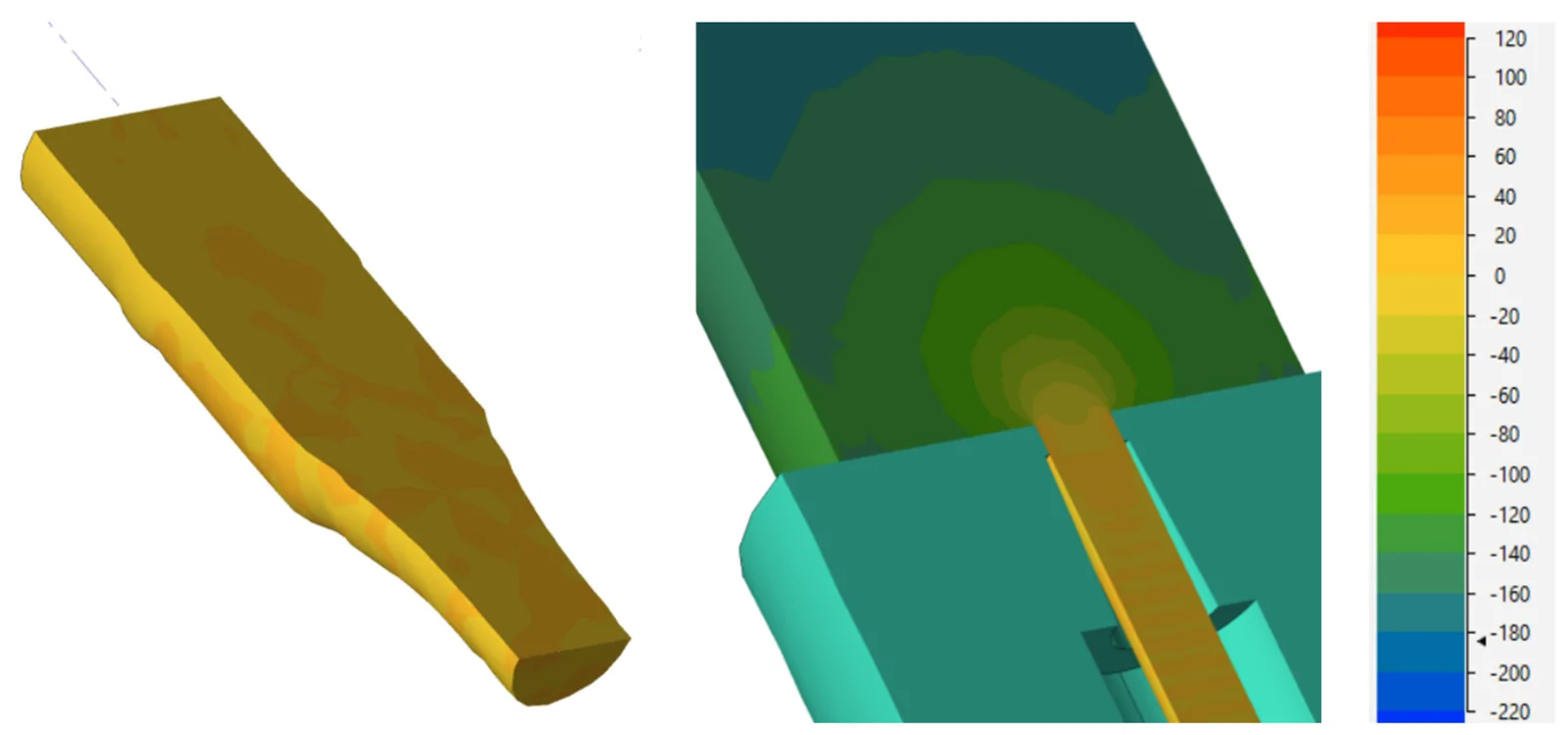
Comparison of the mean stress distribution in hot torsion and extrusion processes obtained from FEM simulations: hot torsion (**left**), extrusion (**right**). A significant difference in the hydrostatic component of the stress state between the analyzed processes is observed.

**Figure 36:**
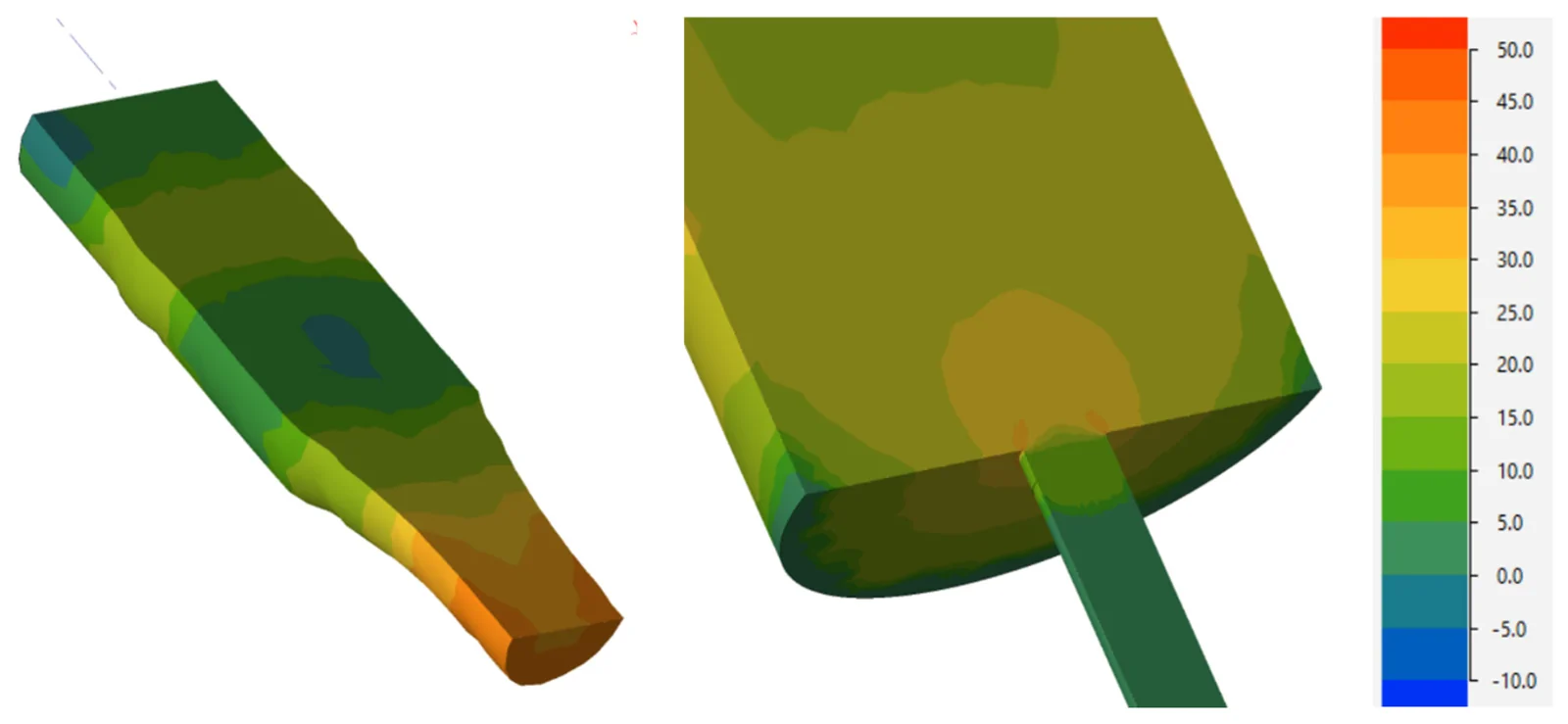
Comparison of the effective stress distribution in hot torsion and extrusion processes obtained from FEM simulations: hot torsion (**left**), extrusion (**right**). Although the range of effective stress values is comparable in the analyzed processes, clear differences in their spatial distribution are observed.

**Figure 37:**
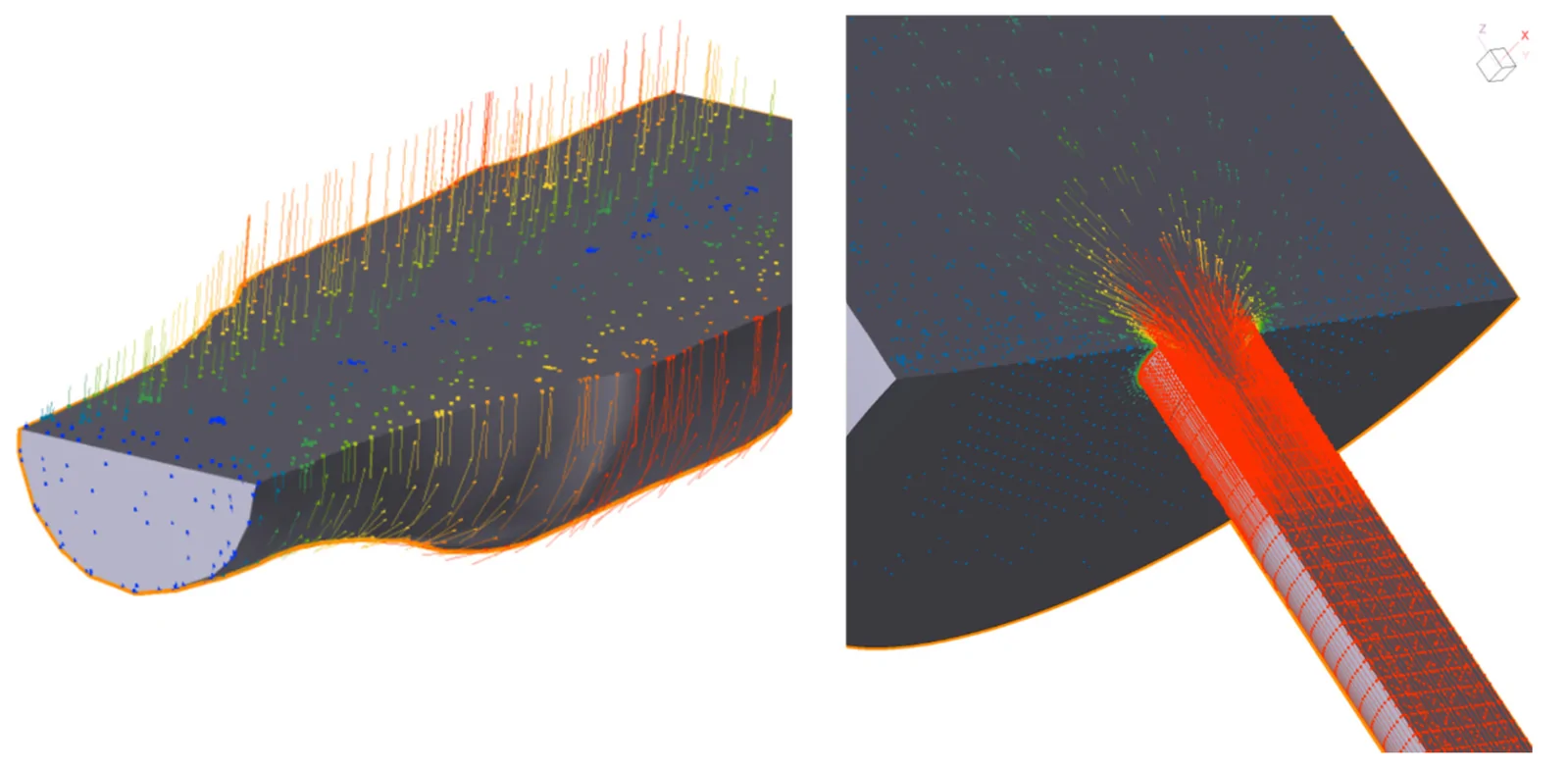
Comparison of the material flow direction and velocity in hot torsion and extrusion processes obtained from FEM simulations: hot torsion (**left**), extrusion (**right**). The points and vectors indicate the local material flow direction and velocity, with vector length corresponding to velocity magnitude. The figure illustrates the different deformation paths; in the torsion case, the vectors represent local material flow velocity rather than angular velocity. These differences may affect grain elongation and morphology.

Additional FEM analysis of the material flow direction, effective stress distribution and mean stress distribution further illustrates the differences in the deformation path and stress state between the two processes (Figure 35, Figure 36 and Figure 37). These results indicate that similar local values of temperature, strain rate and strain do not correspond to an identical thermomechanical state.

### 3.4. Microhardness Results and Analysis

Microhardness measurements were performed on all tested specimens using a Duramin-2500E microhardness tester | EMCO-TEST Prüfmaschinen GmbH, Kuchl, Austria. |at a load of 0.1 kgf (HV0.1). The measurements were performed in areas corresponding to the comparative analysis of the extrusion and torsion processes obtained from finite element modeling, located approximately 0.6r from the axis of the tested specimens, in order to ensure consistency between the experimental measurements and the numerical results. The values presented in the figures represent the arithmetic mean of the obtained results. The differences between individual measurements did not exceed 4%. For each investigated condition, three microhardness measurements were performed in the selected representative area of one specimen. The three measurements were treated as repeated measurements on the same specimen and not as independent experimental replicates. The error bars represent the standard deviation (SD) calculated from the three measurements.

**Figure 38:**
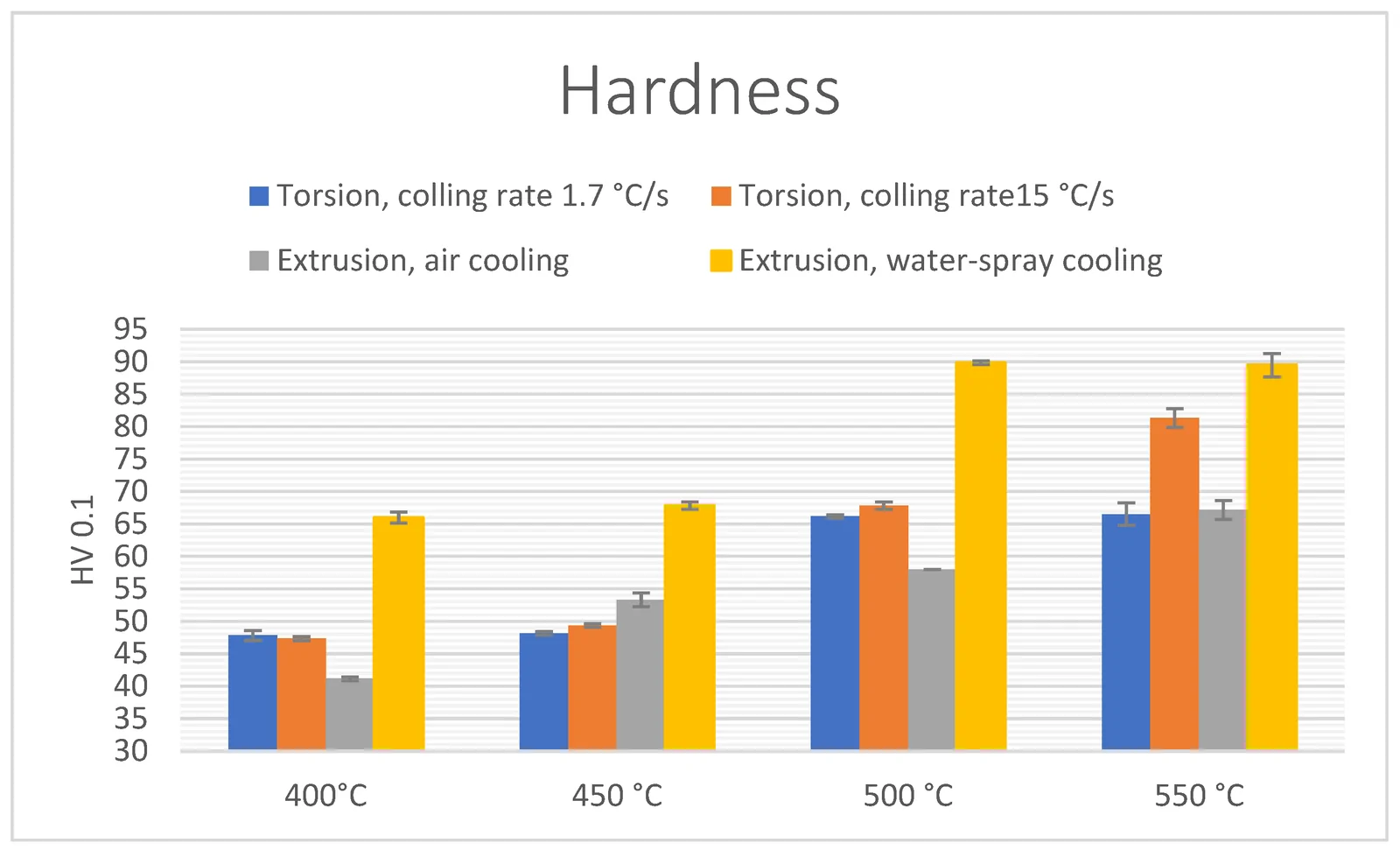
Microhardness test results obtained for EN AW-6060 aluminum alloy specimens after physical modeling in a torsion test and after semi-industrial extrusion.

The microhardness results obtained for specimens subjected to physical modeling in torsion tests, cooled at rates of 1.7 °C/s and 15 °C/s for all tested temperatures are presented in Figure 38. The figure also includes the corresponding specimens after the semi-industrial extrusion process, subjected to two different cooling conditions: air cooling and water mist cooling.

Based on an analysis of the presented test results, an increase in microhardness can be observed as the deformation process temperature rises for all tested specimens subjected to both torsion and extrusion. This trend is particularly pronounced at higher thermoplastic processing temperatures of 500 °C and 550 °C, which can be attributed to the occurrence of precipitation hardening. Microhardness measurements were conducted three weeks after the deformation process, which allowed for the consideration of any aging effects and the stabilization of the material’s properties.

At lower thermoplastic processing temperatures, strain hardening is the dominant mechanism. However, its contribution is partially reduced by the recovery and recrystallization processes occurring during deformation, resulting in relatively lower microhardness values.

When comparing cooling conditions, it was found that higher cooling rates lead to an increase in microhardness values for both deformation processes analyzed. However, specimens obtained after extrusion under semi-industrial conditions and cooled using water mist showed higher microhardness than specimens after torsion and cooling at a rate of 15 °C/s. This difference stems from the slightly more intense cooling achieved during water-mist cooling under semi-industrial conditions (approximately 15–17 °C/s), which contributed to a higher level of supersaturation in the tested aluminum alloy. Furthermore, the temperature measured at the exit of the extrusion press was spatially separated from the die bearing area, where deformation and cooling are most intense, which may have led to a slight underestimation of the actual material temperature during extrusion.

Despite these differences, a comparison of the extrusion process and physical modeling in torsion tests indicates that similar microhardness trends were obtained for comparable thermoplastic processing parameters. The differences between corresponding variants generally did not exceed 10 HV and can be attributed to minor instabilities in process control (such as fluctuations in temperature or strain rate), uncertainties associated with finite element analysis, and differences in the resulting microstructure due to the distinct deformation patterns characteristic of both processes.

## 4. Directions for Future Research

In the long term, the authors plan to further develop the analyzed topic in order to improve the accuracy of physical modeling. Preliminary studies conducted to date (for selected thermoplastic processing parameters) on selected mechanical properties of the tested EN AW-6060 alloy after physical modeling and after the extrusion process have shown good agreement (Table 6).

An analysis of the data presented in Table 6 revealed that the average error in the values of the analyzed mechanical properties of the material after physical modeling and after the extrusion process of the EN AW-6060 alloy was less than 17% (only in some cases did it slightly exceed 20%). This may have been caused by insufficient accuracy of the Hensel–Spittel material model (Equation (2) and Table 2), as a result of which the determined deformation parameters and areas representative for metallographic analyses may have been determined with insufficient accuracy. Furthermore, the controlled cooling process following physical modeling was conducted at a constant rate corresponding to the average cooling rate under semi-industrial conditions. As is well known, in reality, the temperature decay curve during cooling is not linear but rather hyperbolic. Consequently, despite maintaining comparable average cooling rates (during physical modeling and during extrusion on a semi-industrial line), the temperature drop at the beginning of the cooling process under industrial conditions may be greater, which affects the processes of microstructural transformation and formation and, consequently, the mechanical properties of the final product. Additionally, the studies did not measure changes in material temperature within the deformation zone during the extrusion process, and during physical modeling, a constant temperature value was assumed for the EN AW-6060 alloy, whereas in the actual extrusion process it may undergo slight changes, which affects the yield stress—which, in turn, influences the final properties of the tested material, since the course and level of the yield stress determine the microstructural transformation processes and mechanisms that occur.

Due to technical reasons related to the maintenance of the torsion plastometer, additional plots showing the temperature distribution over time could not be generated at this stage. In the future, the authors plan to undertake work aimed at increasing the accuracy of the material model describing the rheological properties of the alloy under study. In addition, the authors plan to conduct studies on temperature changes in the extruded material and to investigate the temperature distribution over time during cooling after the extrusion process. These studies will enable greater accuracy in determining the thermomechanical parameters of the analyzed process, which will improve the accuracy of the physical modeling of the analyzed extrusion process using a torsion test.

## 5. Conclusions

Based on the preliminary studies conducted to assess the possibility of using the hot torsion test for the physical modeling of microstructural evolution during the extrusion process of 16 mm diameter round profiles made of EN AW-6060 aluminum alloy, and following an analysis of the obtained results, the following conclusions were drawn:

1. Finite element method (FEM) numerical modeling performed using QForm Extrusion^®^ software revealed distinct gradients in deformation parameters in the analyzed processes: high-temperature constrained torsion and extrusion. In the torsion test, the values of true strain and true strain rate increased with radial distance, leaving the specimen axis virtually undeformed. The extrusion process was characterized by a slightly lower gradient of strain and strain rate and also with a characteristic increase in the analyzed parameters in the near-surface region.
2. Based on the results of numerical analyses, regions located approximately 0.6 of the specimen radius away were identified as the most representative for comparing the microstructure between torsion and extrusion. In these regions, comparable levels of strain and similar thermal conditions were obtained, which enabled the correlation of the resulting microstructures.
3. Microstructural analysis using light microscopy and EBSD showed that an increase in deformation temperature led to distinct changes in microstructure morphology and an increase in grain size. The EBSD analysis results also confirmed a change in the proportion of recrystallized, partially recrystallized, and deformed grains as process conditions changed.
4. Higher cooling rates, particularly at 500 and 550 °C, partially inhibited post-deformation grain growth by limiting the time available for recrystallization and grain growth. However, the cooling intensity applied was insufficient to completely halt the evolution of the microstructure after deformation.
5. The obtained results confirm that hot torsion testing, combined with the selection of representative regions based on FEM analysis, can serve as a supporting method for modeling microstructural evolution in industrial extrusion processes, provided that the thermomechanical conditions correspond to those occurring in the modeled process.
6. The conducted research is preliminary in nature. Further work will focus on extending the quantitative analysis of microstructural evolution and validating the physical modeling approach for other aluminum alloys and processing conditions.

## Figures and Tables

**Table 1:** Chemical composition of cast billets of EN AW-6060 aluminum alloy, [wt%].

Si	Fe	Cu	Mn	Mg	Cr	Ni	Zn	Ti	Zr	Al
0.43	0.19	0.00	0.02	0.45	0.00	0.00	0.01	0.02	0.00	Balance

**Table 2:** Material coefficients of the Hensel–Spittel equation for the EN AW-6060 alloy used in FEM calculations.

A	m1	m2	m3	m4	m5	m7	m8	m9
198	−0.005	−0.148	0.13	−0.020	0.0028	0	0	0

**Table 3:** Main parameters of the thermoplastic forming process used in the studies.

Strain temperature, [°C]	400, 450, 500, 550
True strain, [-]	5
Strain rate, [s^−1^]	5
Cooling rate, [°C/s]	1.7 (variant 1) and 15 (variant 2)

**Table 4:** Comparison of local thermomechanical parameters in the representative regions of the hot torsion and extrusion processes.

Parameter	Hot Torsion, (0.6r Region)	Extrusion, Corresponding Region
Local temperature, °C	503.5–504.5	515–532
True strain	3.2–3.6	3.3–3.8
Strain rate, s^−1^	4.5–5	4.2–5

**Table 5:** Quantitative grain-size characteristics obtained from EBSD analysis.

Deformation Process	Temperature	Mean Grain Diameter	Standard Deviation
	[°C]	[µm]	[µm]
Extrusion	450	72.0	39.8
Extrusion	550	84.8	37.3
Torsion	450	21.6	15.57
Torsion	550	48.7	21.2

**Table 6:** Selected mechanical properties of the EN AW-6060 alloy after physical modeling and after the extrusion process.

Thermoplastic Forming Temperature	Cooling Rate After Deformation	Physical Modeling—Torsion Test			Extrusion Under Semi-Industrial Conditions		
		Yield Stress, YS	Ultimate Tensile Strength, UTS	Elongation, A_10_	Yield Stress, YS	Ultimate Tensile Strength, UTS	Elongation, A_10_
[°C]	[°C/s]	[MPa]	[MPa]	[%]	[MPa]	[MPa]	[%]
550	15	111	233	35.7	98.6	204.6	27.1
550	1.7	101	222	36.5	81.6	189.7	31.7
500	15	109	226	36.1	88.0	192.9	29.0
500	1.7	101	215	31.1	78.0	171.7	28.7

## Data Availability

The original contributions presented in this study are included in the article. Further inquiries can be directed to the corresponding author.
